# Factors associated with admission before the day of elective major surgery: an analysis of data from the UK Perioperative Quality Improvement Programme

**DOI:** 10.1016/j.bja.2025.10.064

**Published:** 2025-12-31

**Authors:** Bo Hou, Tom Salih, James Bedford, Adam Hunt, Duncan Wagstaff, Arun Sahni, Eleanor Warwick, PQIP delivery team, Suneetha Ramani Moonesinghe, the PQIP Collaborative

**Affiliations:** 1 National Institute for Health Research Central London Patient Safety Research Collaboration (PSRC), University College London Hospitals NHS Foundation Trust, London, UK; 2 Research Department of Targeted Intervention, Division of Surgery and Interventional Science, University College London, London, UK; 3 Department of Anaesthesia and Perioperative Medicine, University College London Hospitals NHS Foundation Trust, London, UK; 4 Centre for Research and Improvement, Royal College of Anaesthetists, London, UK; 5 Department of Anaesthesia and Perioperative Medicine, Broomfield Hospital, Mid and South Essex NHS Trust, Chelmsford, UK; 6 Department of Anaesthesia and Perioperative Medicine, Kings College Hospital NHS Foundation Trust, London, UK

**Keywords:** day surgery admission, hospital length of stay, hospital capacity, Natioanl Health Service, patient safety, Perioperative Quality Improvement Programme

## Abstract

**Background:**

Hospital capacity within the UK National Health Service is under unprecedented pressure. Reducing admission before the day of elective surgery might be an opportunity to reduce bed occupancy and improve patient flow, safety, and experience.

**Methods:**

This was a secondary analysis of data from the UK Perioperative Quality Improvement Programme (PQIP), a prospective observational cohort study. Primary outcome was admission to hospital before the day of surgery, and secondary outcome was prolonged postoperative length of stay. We used mixed-effects multivariable regression modelling to evaluate associations with patient, case, and hospital characteristics.

**Results:**

We included 54 267 patients undergoing major elective surgery between December 2016 and February 2024, of whom 9124 (17%) were admitted before their surgical date. In adjusted analysis, predictors of early admission included increased straight-line distance from home to hospital (odds ratio 1.93, 95% confidence interval [1.59–2.35] for distance >64 km compared with <6 km), hypoalbuminaemia, anaemia, raised creatinine, poorly controlled diabetes mellitus, use of bowel preparation, failure to complete preassessment, and surgical specialty. Likelihood of early admission was clustered by hospital (intraclass correlation coefficient=0.527). Postoperative length of stay was associated with identifiable case-level factors that explained most of the observed variation (intraclass correlation coefficient=0.063).

**Conclusions:**

Multiple risk factors for admission before day of surgery could be addressed through policy or local quality improvement. Unexplained variation at the hospital level might reflect local culture and process, which should be explored in future research. We recommend exploring hotel-type accommodation as a potentially cost-effective alternative to preoperative inpatient admission for suitable patients.

**Editor’s key points**

• Given mounting pressures on hospital capacity within the UK National Health Service, reducing admission before the day of elective surgery and hospital length of stay are opportunities to reduce bed occupancy and improve patient care.
• This secondary analysis of data from the UK Perioperative Quality Improvement Programme (PQIP) analysed admission to hospital before the day of surgery as a primary outcome, and prolonged postoperative length of stay as a secondary outcome.
• Of patients undergoing elective major surgery, 17% were admitted in the 24 h before the day of surgery, which was associated with greater distance between home and hospital, inadequate preoperative preparation, and local cultures.
• These risk factors for early hospitalisation could potentially be addressed through policy and local quality improvement to improve patient flow and reduce hospital occupancy.

There is an acknowledged lack of bed capacity in the UK National Health Service (NHS), with hospitals running at >95% occupancy over the winter of 2024/2025, against a target of <92%.^1^ There is increasing concern over ‘corridor care’ becoming normalised, that is, emergency patients who require admission being treated in corridors for hours or even days because no inpatient beds are available.^2^ Moreover, an estimated 6.4 million patients are currently on NHS waiting lists in England,^3^ ∼20% of whom are waiting for surgery. Tackling this requires an increase in surgical productivity, which is dependent on factors including hospital bed capacity.^4^ Lack of bed capacity is a significant factor in cancellation of elective surgery.^5^

The NHS has a comparatively high rate of bed utilisation after elective procedures,^6^ despite a focus on maximising day surgery capability over the past few years.^7^ Bed capacity is additionally reduced by pressure from nonsurgical services.^8^ The NHS Long Term Plan includes a commitment to reduce the proportion of inpatient care.^9^^,^^10^

Despite initiatives to reduce hospital admissions, it is accepted that some procedures require a postoperative inpatient stay because of either surgical or patient-related factors. The duration of elective surgical admissions has reduced over time as a result of efforts to improve efficiency and initiatives such as enhanced recovery pathways.^11^ Modern surgical and anaesthetic techniques also facilitate earlier discharge from hospital.^12^^,^^13^

Previous trends of admitting patients before the day of surgery for ‘optimisation’ have largely been superseded through the implementation of preoperative assessment and the knowledge that patients are at lower risk of complications if comorbidities such as diabetes mellitus and anaemia are optimised before hospital admission.^13^ Despite this, some patients continue to be admitted before the day of surgery. It is unclear what proportion of these early admissions are clinically essential or could be avoided through better preoperative preparation or alternative models of care. Nonclinical reasons for early admission to hospital might include distance between place of residence and hospital of treatment or other factors associated with being unable to attend a treatment centre in a timely way. Not only is there an efficiency incentive for reducing unnecessary hospitalisation, but there is also a potential patient safety incentive, with broad acceptance that hospital admission carries risk.^14^

This study aimed to evaluate factors which were associated with admission before the day of elective major surgery using a large national cohort of patients treated in England and Wales. In addition, we also evaluated factors associated with prolonged length of stay (LOS) after surgery. We hypothesised that modifiable factors could be identified that were associated with admission before surgery and prolonged LOS. We specifically hypothesised that increased distance to hospital would be associated with more time in hospital, both as a result of earlier preoperative admission (for travel and logistic reasons) and prolonged postoperative LOS (either as a marker of additional perioperative risk or by influencing clinical decisions to discharge patients). The purpose of the study was to inform future recommendations to providers and policy makers on opportunities to improve utilisation of hospital beds around the time of surgery.

## Methods

This was a retrospective secondary analysis using data from the Perioperative Quality Improvement Programme (PQIP^15^), a prospective observational cohort study of patients aged 18 yr or older undergoing major, planned noncardiac surgery in UK NHS hospitals; the study protocol and details on data acquisition have been described.^16^ PQIP was approved by the Health Research Authority (London–Surrey Research Ethics Committee REC reference number: 16/LO/1827). The findings reported conformed to the Strengthening the Reporting of Observational Studies in Epidemiology statement.^17^

### Outcomes and covariates

The primary outcome was whether the patient was admitted before the day of surgery, defined as a binary variable by whether the patient was admitted in the 24 h before the surgical date. Our secondary outcome was prolonged postoperative LOS, which was defined as a binary variable by postoperative LOS over the 90th percentile for that surgical specialty.^18^

We included patients with a valid postcode from England and Wales recruited between December 2016 and February 2024 (*N*=54 267). We excluded patients admitted >1 day before the day of surgery, to exclude patients who were admitted acutely and then waited as an inpatient for an elective surgery appointment to become available. Additionally, patients living >200 km from the hospital (*n*=700, comprising 1.3% of the 54 267 patient cohort) were also excluded, as we considered it infeasible that these patients would be able to travel from home on the day of surgery. We excluded patients who did not have a discharge date and patients who died in hospital.

We chose variables in the PQIP dataset that had a plausible causal relationship with our primary and secondary outcomes. These included demographic factors, clinical risk factors, and processes of care. Demographic and clinical factors included distance between home and hospital, age, sex, and body mass index (BMI) categorised as low, normal/high, or very high, history of cerebrovascular disease, dementia, diabetes mellitus, American Society of Anesthesiologists (ASA) physical status categorised as 1–2 *vs* 3–5, and Rockwood clinical frailty score categorised as not frail (1–4), mild (5), or moderate (6–9). Investigations included serum sodium, creatinine, white cell count, haemoglobin, and heart rate, which were coded as abnormal or normal using threshold values listed in Table 1. Relevant perioperative processes and metrics were also included such as surgical indication (cancer *vs* noncancer), urgency, complexity and speciality, whether a patient was on an enhanced recovery pathway, preoperative assessment status, planned postoperative destination, whether the patient received bowel preparation, and the day of the week on which surgery took place (Table 2). Finally, we accounted for rurality of patients’ Lower-Layer Super Output Areas (LSOAs) and the NHS geographical region of the treating hospital (e.g. London, North East, East of England, etc).

**Table 1:** Descriptive statistics by day of surgery admission (*n*=52 213). HbA1c, haemoglobin A1c; IQR, interquartile range.

	Day of surgery admission yes/no	
	Yes (*n*=44 523)	No (*n*=7690)
	Median (IQR)	Median (IQR)
Straight-line distance (km)	12.1 (5.6–22.5)	16.9 (7.6–35.3)
Age (yr)	66 (56–73)	67 (57–74)

	N	%	N	%
Sex				
Female	19 915	44.7	3455	44.9
Male	24 608	55.3	4235	55.1
Body mass index				
Low	424	1	125	1.6
Normal/high	42 302	95.5	7278	95
Very high	1557	3.5	258	3.4
Urgency of surgery				
Elective	40 111	92.6	6599	87.1
Expedited	3193	7.4	980	12.9
Cancer diagnosis or in remission for <5 yr				
No	14 628	33.9	2073	27.5
Yes	28 460	66.1	5464	72.5
Sodium				
Normal (133–146 mM)	43 512	97.7	7438	96.7
Abnormal	1011	2.3	252	3.3
Creatinine				
Normal (male 59–104 μM; female 45–84 μM)	33 002	74.1	5371	69.8
Abnormal	11 521	25.9	2319	30.2
Albumin				
Normal (35–50 g L^−1^)	41 766	93.8	7075	92
Abnormal	2757	6.2	615	8
White cell count (4–11×10^9^ L^−1^)				
Normal	41 791	93.9	7193	93.5
Abnormal	2732	6.1	497	6.5
Haemoglobin				
Normal (male 13.6–17.7 g dl^−1^; female 12.1–15.1 g dl^−1^)	22 194	49.8	3260	42.4
Abnormal	22 329	50.2	4430	57.6
Heart rate				
Normal (60–100 beats min^−1^)	39 199	88	6722	87.4
Abnormal	5324	12	968	12.6
History of cerebrovascular disease				
No	41 501	96.3	7159	94.9
Yes	1595	3.7	384	5.1
Dementia				
No	42 816	99.4	7502	99.5
Yes	272	0.6	39	0.5
Diabetes mellitus				
No diabetes mellitus	37 592	89.7	6323	88.3
T1 and HbA1c≤8.5%	103	0.2	35	0.5
T1 and HbA1c>8.5%	63	0.2	18	0.3
T2 and HbA1c≤8.5%	3264	7.8	586	8.2
T2 and HbA1c>8.5%	874	2.1	200	2.8
ASA physical status				
1 and 2	30 407	70.7	4466	59.3
3–5	12 615	29.3	3069	40.7
Rockwood clinical frailty score				
Not frail 1–4	25 819	58	4279	55.6
Mild 5	760	1.7	172	2.2
Moderate 6–9	425	1	119	1.5
Not known	17 519	39.3	3120	40.6
Rural urban classification				
Rural	9956	22.4	1552	20.2
Urban	34 567	77.6	6138	79.8
Index of multiple deprivation 2019				
Most deprived quintile	6019	14	884	14.2
2nd most deprived	7859	18.2	1183	19
3rd most deprived	9558	22.2	1306	21
2nd least deprived	9707	22.5	1424	22.9
Least deprived quintile	9981	23.1	1415	22.8
Provider regions				
East Midlands	1393	3.1	83	1.1
East of England	6600	14.8	598	7.8
London	7212	16.2	2638	34.3
North East	2176	4.9	250	3.3
North West	5824	13.1	1250	16.3
South East	6866	15.4	443	5.8
South West	5881	13.2	112	1.5
Wales	1190	2.7	1434	18.6
West Midlands	4422	9.9	719	9.3
Yorkshire and The Humber	2959	6.6	163	2.1

	Admitted on day of surgery		Admitted before day of surgery		Row total
	*N*	Row %	*N*	Row %	
Specialty					
Abdominal—hepatobiliary	1941	65.3	1031	34.7	2972
Abdominal—lower gastrointestinal	19 418	91.1	1894	8.9	21 312
Abdominal—upper gastrointestinal	2307	76.9	693	23.1	3000
Abdominal—other	1096	89.8	124	10.2	1220
Burns and plastics	1124	91.8	100	8.2	1224
Gynaecology	2155	90.8	219	9.2	2374
Head and neck	678	65.6	355	34.4	1033
Not recorded	1329	91.0	132	9.0	1461
Orthopaedics	2421	92.7	192	7.3	2613
Spinal	831	68.7	378	31.3	1209
Thoracics	3138	61.7	1949	38.3	5087
Urology	7400	93.2	539	6.8	7939
Vascular	685	89.1	84	10.9	769
Surgery complexity	*N*	%	*N*	%	
Complex/complex major	39 030	87.7	5903	76.8	
Major/not known	5493	12.3	1787	23.2

**Table 2:** Processes of care according to day of surgery admission.

Process measure	Admitted on day of surgery		Admitted before day of surgery	
	*N*	%	*N*	%
Day of surgery				
Monday	9078	20.4	1317	17.1
Tuesday	10 804	24.3	1968	25.6
Wednesday	10 065	22.6	2043	26.6
Thursday	9002	20.2	1662	21.6
Friday	5289	11.9	675	8.8
Saturday	165	0.4	17	0.2
Sunday	120	0.3	8	0.1
Enhanced recovery pathway				
No	10 884	24.4	2277	29.6
Yes	25 607	57.5	4171	54.2
Not known	8032	18	1242	16.2
Preoperative assessment				
Yes	42 525	99.2	7034	96.1
No	349	0.8	289	3.9
Patients administered bowel preparation				
Yes	11 691	26.3	1625	21.1
No	19 069	42.8	3171	41.2
Not applicable	13 763	30.9	2894	37.6
Planned postoperative destination				
Ward care	23 350	54.2	3050	40.4
Level 1 care	7827	18.2	1124	14.9
Level 2 care	10 358	24	2952	39.1
Level 3 care	1557	3.6	415	5.5

For the prolonged postoperative LOS analysis, we additionally included factors demonstrated to have an association with LOS, including duration of surgery, Clavien–Dindo grade of complication, highest pain score during recovery stay, postoperative nasogastric tube use, and delivery of drinking, eating, and mobilising (DrEaMing) within 24 h of surgery ending.^19^

### Distance travelled from home to hospital

Patients’ residential LSOA (a geographic unit in England and Wales used for reporting small-area statistics) were generated for anonymity from aggregating patients’ home postcodes collected in the PQIP dataset. Each LSOA, a UK census geography unit, included between ∼1000 and 3000 individuals.^20^ Coordinates of the population weighted centroid for patients’ respective LSOA were geocoded using Office for National Statistics Population Weighted Centroids.^21^ Coordinates of hospital sites were generated using full postcodes obtained from NHS Choices, and geocodes were found using the Open Geography Portal.^22^ Straight-line distances travelled from home to hospital were calculated based on these two coordinates.

As we hypothesised that the relationship between distance to hospital from home and outcomes was not likely to be linear, straight-line distance was modelled using restricted cubic splines with five knots, with the position of each knot at 5, 27.5, 50, 72.5, and 95 percentiles, respectively.^23^^,^^24^

### Statistical analysis

We described baseline variables according to whether the patient was admitted before the day of surgery. Frequency and row percentages are shown, and for continuous variables, median and interquartile range are included. For multivariate analyses, a mixed-effects two-level random intercept logistic regression was used to account for clustering by hospital sites. Odds ratio (OR), 95% confidence interval (CI) and exact *P*-values were reported. After the mixed-effects logistic regression model, a Wald test was done to assess the joint statistical significance of distance to hospital variable. A Stata package (xbrcspline, StataCorp, College Station, TX, USA) was used to display differences in the predicted ORs and 95% CI when setting reference value to knot 1 of distance variable. Age was centred on its mean. A squared term of centred age was included to capture the commonly observed U-shaped or inverted U-shaped nonlinear relationship between age and health outcomes.^25^ To look into variations in outcome between different hospital sites, intraclass correlation coefficient (ICC) was estimated and reported after mixed-effects logistic regression.^26^ Robust standard errors were used to account for heteroskedasticity.

Complete case analyses were conducted. Our analytical sample for the day of surgery admission analysis started with 52 213 patients initially and ended with 48 369 patients in our fully adjusted multilevel multivariable logistic model. Therefore 3844 (7.4%) were excluded for this specific analysis in our fully adjusted model because of missingness in one or more covariates. Similarly, the cases or percentage for our analysis on prolonged postoperative LOS analysis was 2761 (5.4%).

Our primary outcome and key exposure variable of straight-line distance to hospital had no missing data. There were small amounts of missing data in our confounders, but none >5%. Variables with >10% missing were frailty, whether or not the patient was on an enhanced recovery pathway, white cell count, and serum albumin.

For the main analyses, before applying complete case analysis, missing values for white cell count and serum albumin were categorised as normal. Our rationale was grounded in clinical practice. These tests were generally not routine for all elective surgeries; they were ordered when a clinician suspects an abnormality. We therefore reasoned that the absence of a test result likely reflects an absence of clinical concern. We acknowledge that this could introduce bias, meaning the reported effects for these variables were likely conservative estimates.

A formal power calculation was not conducted before the analysis; however, with 7690 outcome events for the primary outcome and 5019 for the secondary outcome, both models were sufficiently powered and not at risk of overfitting, comfortably meeting the standard ‘one in ten’ events per variable rule.^27^^,^^28^

Analyses were conducted using Stata 18 (StataCorp, 2023) and R Statistical Software (v4.3.2; R Core Team, 2023, R Foundation for Statistical Computing, Vienna, Austria). Statistical significance was defined at a two-sided *P*-value <0.05.

### Sensitivity analysis

Several sensitivity analyses were conducted. Firstly, we adjusted for socioeconomic deprivation using the index of multiple deprivation 2019.^29^ This was done on the sample of all patients who lived in England (therefore excluding those in Wales and Scotland). Secondly, we evaluated different methods for deriving and classifying the distance to hospital variable in our models including using previously published categorisations.^30^ Thirdly, multiple imputation by chained equations was used to address missing data (see Supplementary material, Section S8 for more detail).

## Results

### Descriptive statistics

**Fig 1:**
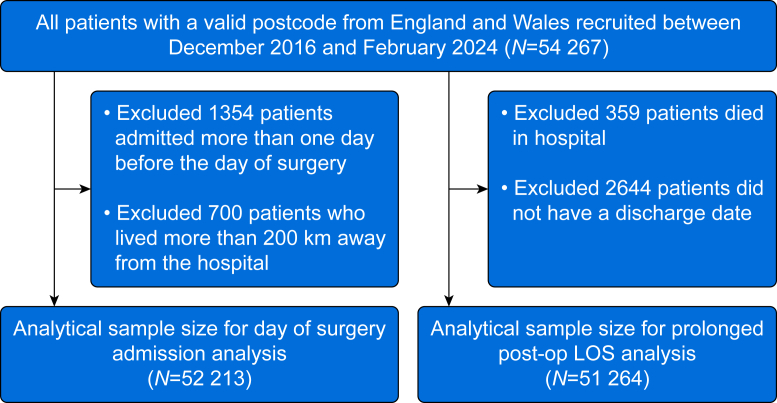
Study flowchart. LOS, length of stay.

Of the 54 267 patients in our cohort, 9124 (16.8%) were admitted to hospital before the day of surgery. The study flow is described in Figure 1. The median distance to hospital travelled for the cohort was 12.3 km (interquartile range 5.7–23.2 km) for patients who had surgery on the same day of admission, and 16.9 km (interquartile range 7.6–35.9 km) for patients who were admitted before the day of surgery (Supplementary material, Section S1). Patients treated in the South East, East of England, and Wales were more likely to be treated out of region than patients treated in London, the North East, North West, Midlands, or South West. (Supplementary Table 2). There were some differences in baseline characteristics according to whether or not the patients were admitted before the day of surgery (Table 1). Patients who were admitted before the day of surgery had higher rates of some abnormal preoperative tests, for example creatinine (30.2% *vs* 25.9%), higher frequency of a cancer diagnosis (72.5% *vs* 66.1%), and were more likely to be undergoing expedited (*vs* elective) surgery (12.9% *vs* 7.4%).

### Main results

After controlling for all covariates in a mixed-effects logistic regression model (Table 3), straight-line distance from home to hospital was associated with admission before the day of surgery (*P*<0.001). As distance between home and hospital increased, so did the OR of being admitted before the day of surgery, in a nonlinear fashion, with significantly increased odds of >22.3 km distance.

**Table 3:** Distance to hospital and admission before day of surgery—all patients England and Wales in PQIP, mixed-effects logistic regression, odds ratio reported (*N*=48 369). CI, confidence interval; GI, gastrointestinal; HbA1c, haemoglobin A1c; HPB, hepatobiliary; OR, odds ratio. † Wald test was done to assess the joint statistical significance of distance to hospital variable. ∗ *P* < 0.05, ∗∗ *P* < 0.01, ∗∗∗ *P* < 0.001.

	OR	*P*-value	95% CI
Distance to hospital (knot 1–1.91 km as reference)^†^		(<0.001)	
Knot 2–6.36 km	1.000		(0.890–1.140)
Knot 3–12.71 km	1.120		(0.950–1.320)
Knot 4–22.30 km	1.340		(1.140–1.580)
Knot 5–62.57 km	1.930		(1.590–2.350)
Patient characteristics			
Centred age	1.008∗∗	(0.004)	(1.002–1.013)
Centred age squared	1.000∗∗∗	(<0.001)	(1.000–1.000)
Male sex	1.092	(0.098)	(0.984–1.212)
Surgical specialties and complexity			
Ref: abdominal—lower GI, complex/complex major			
Abdominal-HPB, complex/complex major	3.174∗∗	(0.004)	(1.434–7.026)
Abdominal—Hepatobiliary, major/not known	2.907	(0.126)	(0.740–11.412)
Abdominal—Lower GI, major/not known	1.916∗	(0.035)	(1.045–3.513)
Abdominal—Upper GI, complex/complex major	4.028∗	(0.029)	(1.152–14.088)
Abdominal—Upper GI, major/not known	2.091∗	(0.046)	(1.014–4.311)
Abdominal—Other, complex/complex major	1.421	(0.190)	(0.840–2.405)
Abdominal—Other, major/not known	1.278	(0.657)	(0.432–3.787)
Burns and plastics, complex/complex major	2.948	(0.364)	(0.286–30.389)
Gynaecology, complex/complex major	0.801	(0.713)	(0.245–2.619)
Gynaecology, major/not known	1.444	(0.766)	(0.128–16.258)
Head and neck, complex/complex major	18.861∗∗∗	(<0.001)	(7.092–50.159)
Head and neck, major/not known	62.497∗∗	(0.005)	(3.467–1126.566)
Orthopaedics, complex/complex major	0.556	(0.225)	(0.215–1.436)
Spinal, complex/complex major	4.957∗∗∗	(<0.001)	(2.086–11.779)
Spinal, major/not known	85.384∗∗∗	(<0.001)	(14.335–508.563)
Thoracics, complex/complex major	12.017∗∗∗	(<0.001)	(4.283–33.713)
Thoracics, major/not known	14.506∗∗∗	(<0.001)	(4.987–42.195)
Urology, complex/complex major	1.45	(0.367)	(0.647–3.247)
Urology, major/not known	1.31	(0.459)	(0.641–2.679)
Vascular, complex/complex major	4.050∗∗	(0.004)	(1.576–10.412)
Urgency of surgery: ref elective			
Expedited	1.358∗	(0.034)	(1.023–1.803)
Cancer diagnosis or in remission for <5 yr: ref No			
Yes	1.244	(0.058)	(0.992–1.559)
Enhanced recovery: ref Yes			
No	0.901	(0.532)	(0.651–1.248)
Unknown	0.847	(0.138)	(0.680–1.055)
Preop-assessment: ref Yes			
No	1.77	(0.143)	(0.825–3.797)
Sodium: ref normal			
Abnormal	1.062	(0.607)	(0.844–1.336)
Creatinine: ref normal			
Abnormal	1.134∗∗∗	(0.001)	(1.052–1.222)
Albumin: ref normal			
Abnormal	1.298∗∗∗	(0.001)	(1.119–1.505)
White cell count: ref normal			
Abnormal	1.082	(0.414)	(0.895–1.308)
Haemoglobin: ref normal			
Abnormal	1.343∗∗∗	(<0.001)	(1.227–1.470)
Heart rate: ref normal			
Abnormal	0.972	(0.621)	(0.871–1.086)
Cerebrovascular disease: No			
Yes	1.055	(0.566)	(0.878–1.269)
Dementia: No			
Yes	0.807	(0.275)	(0.550–1.186)
Diabetes mellitus: ref No diabetes mellitus			
T1 and HbA1c≤8.5	2.674∗∗	(0.008)	(1.293–5.529)
T1 and HbA1c>8.5	2.636∗∗	(0.008)	(1.282–5.421)
T2 and HbA1c≤8.5	1.040	(0.579)	(0.905–1.195)
T2 and HbA1c>8.5	1.435∗∗	(0.002)	(1.145–1.798)
ASA physical status: ref 1 and 2			
3–5	1.170∗	(0.037)	(1.010–1.357)
Planned post-op destination: ref ward care			
Level 1 care	1.291	(0.130)	(0.928–1.797)
Level 2 care	2.534∗∗∗	(<0.001)	(1.863–3.446)
Level 3 care	3.861∗∗∗	(<0.001)	(2.197–6.785)
Bowel prep: ref No			
Yes	2.435∗∗∗	(<0.001)	(1.614–3.674)
Frailty: ref not frail 1–4			
Mild 5	1.373∗	(0.038)	(1.018–1.851)
Moderate 6–9	1.844∗∗	(0.002)	(1.257–2.706)
Not done or not known	1.426∗	(0.015)	(1.073–1.895)
Body mass index: normal/high			
Low	1.289	(0.143)	(0.917–1.811)
Very high	0.913	(0.479)	(0.709–1.175)
Day of surgery: ref Tuesday			
Fri	0.88	(0.437)	(0.638–1.214)
Mon	0.724∗	(0.013)	(0.561–0.935)
Sat	1.627	(0.427)	(0.490–5.410)
Sun	0.493	(0.218)	(0.160–1.520)
Thu	1.187	(0.209)	(0.909–1.549)
Wed	1.119	(0.316)	(0.898–1.394)
Provider region: East of England			
London	2.723∗	(0.044)	(1.029–7.206)
North East	1.99	(0.310)	(0.526–7.526)
North West	8.242∗∗∗	(<0.001)	(2.997–22.672)
South East	1.092	(0.847)	(0.445–2.682)
South West	0.617	(0.330)	(0.234–1.630)
Wales	40.924∗∗∗	(<0.001)	(5.267–317.996)
East Midlands	0.5	(0.493)	(0.069–3.622)
West Midlands	1.431	(0.622)	(0.344–5.951)
Yorkshire and The Humber	2.626	(0.175)	(0.652–10.582)
Rurality: ref rural			
Urban	1.049	(0.349)	(0.949–1.160)
Observations	48 369		
Intraclass correlation coefficient	0.527		

Increasing age, abnormal preoperative tests (low albumin and high creatinine, higher ASA physical status, poor diabetes mellitus control, and mild or moderate frailty) were independently associated with risk of admission before the day of surgery (Table 3). Some types and magnitudes of surgery were associated with admission before the day of surgery, particularly complex head and neck and thoracic surgery. Some processes of care were also predictive: in particular, patients who required bowel preparation were more likely to be admitted early. Patients were less likely to be admitted before the day of surgery if their procedure was on a Monday. Patients treated in Wales, London, and the North West of England were more likely to be admitted before the day of surgery.

ICC from the mixed-effects logistic regression model was 0.527. This suggests 52.7% of the variation in the outcome of whether a patient was admitted before the day of surgery was attributed to unmeasured differences at the hospital level. A caterpillar plot of hospital-level variation from the fully adjusted model was also plotted and shown in Supplementary Figure 3.

Our secondary outcome was prolonged LOS defined as >90% centile for that specialty; patient characteristics classified according to this outcome are described in Supplementary Table 4. Although distance to hospital was associated with prolonged postoperative LOS (*P*=0.048), coefficients for each knot were not significant compared with knot 1(e.g. OR for knot 5 was 1.14, 95% CI [0.95–1.37]), and other, well-recognised patient and surgical factors were more significant. For example, worse long-term health (e.g. moderate frail, OR=2.105, 95% CI [1.632–2.716]), increasingly complex surgery (e.g. duration of surgery >3 h, OR=2.770, 95% CI [2.285–3.358]), and the occurrence of postoperative complications (e.g. grade 3 and above, OR=35.081, 95% CI [29.334–41.953]), and failure to adhere to processes known to be associated with better outcomes, such as DrEaMing within 24 h (OR=0.420, 95% CI [0.363–0.486]) (Supplementary material, Section S5). ICC from the mixed-effects logistic regression was 0.063. This suggested 6.3% variation in outcome was attributed to unmeasured differences at hospital site level.

### Sensitivity analyses

We analysed data from patients who lived in England including the 2019 index of multiple deprivation quintiles, which did not substantially change our findings (Supplementary material, Section S6). Our results also held with further sensitivity tests that operationalised distance to hospital variable in different forms (Supplementary material, Section S7). Results from multiple imputation analyses also supported our findings above (Supplementary material, Section S8).

## Discussion

Inpatient capacity in the NHS is under unprecedented pressure from both elective and emergency services. Policy efforts to improve resource utilisation in elective care include promotion of ambulatory care, preoperative assessment, and optimisation to reduce complications and postoperative LOS, and embedding enhanced recovery principles, which include day of surgery admission. Additionally, billions of pounds have been spent on development of elective surgical hubs which provide ring-fenced capacity for elective care. Studying almost 55 000 adult patients undergoing major elective surgery, we report that 17% are admitted in the 24 h before the day of surgery, that there are clear associations with modifiable factors, and that unmeasured hospital-level factors might also be important. Reducing the proportion of patients who are admitted unnecessarily before the day of surgery could represent an ‘easy win’ for a health service under severe pressure, but this has not been prioritised by NHS policy in recent years.

Admission before the day of surgery has both cost and safety risks. Reducing unnecessary time in hospital is important to reduce the risk associated with hospitalisation for patients^14^ and to reduce the knock-on harms associated with high bed occupancy,^31^ poor flow through hospitals,^32^ and therefore a risk of delayed care, particularly for emergency patients.^33^^,^^34^ In the UK, day of surgery admission has been explored far less than postoperative LOS and could represent a previously under-investigated opportunity for quality improvement. The NHS has also focused on increasing day surgery rates, latterly by very small percentages. Moving from 80% to 85% compliance with day surgery standards nationally, in the group of around 3.5 million patients eligible for ambulatory care,^35^ would result in ∼175 000 bed days saved. By contrast, an estimated 1.5 million patients undergo inpatient surgery in the NHS every year. If just 10% of these individuals are admitted unnecessarily before surgery, compared with the 17% pre-day of surgery admission incidence in our analysis, this represents 150 000 wasted bed days, which given the findings of our mixed-effects modelling are likely to be disproportionately concentrated in a subset of institutions. Our data therefore indicate that similar gains in available bed days might be possible through prioritising day of surgery admission and the processes which support it; improving those processes (e.g. preoperative assessment and optimisation) would also support improved day surgery rates.

We explored LOS as a secondary analysis, to test two hypotheses: that patients having surgery further from home have otherwise unmeasured increased risk of adverse outcomes; or that patients who had longer distances to travel upon discharge are treated differently, and clinicians opt to keep them in hospital for longer, to reduce the risk of readmission. Our findings were consistent with previous studies that have explored predictors of increased LOS, with the occurrence of postoperative complications being the overwhelmingly most important variable, and our prior hypotheses were not supported. However, this analysis yielded an important, unexpected finding: that very little (6.3%) of the variance in prolonged LOS was not explained by our model, compared with the 52.7% of variance in pre-day of surgery admission which was attributable to unmeasured hospital-level differences. Again, this might reflect the success of clinical and academic endeavours to understand the reasons for prolonged LOS and address them; similar focus on pre-day of surgery admission might yield meaningful efficiency savings.

Therefore, looking to solutions, it is important to explore the reasons underpinning some of the associations we found. Patients undergoing complex thoracic or head and neck surgery were particularly likely to be admitted before the day of surgery, which may be related to specific behaviours of teams in tertiary or specialist centres. Patients receiving bowel preparation were also more likely to be admitted before the day of surgery after accounting for other factors; however, we routinely treat patients requiring bowel preparation for colonoscopy as outpatients, so that in and of itself should not require hospitalisation. These trends could have evolved for unavoidable clinical reasons, or they could reflect modifiable practice. Local audit, review, and qualitative evaluation are required to explore these apparent inefficiencies and address them; for example, in one study of thoracic surgery in Ireland, day of surgery rate for thoracic patients increased from 10% to 75% through implementation of a quality improvement programme using Lean Six Sigma principles.^12^

Several markers of inadequate preoperative care were also associated with admission before the day of surgery, including failure to attend preoperative assessment, and the presence of anaemia or poorly controlled diabetes mellitus. Modern guidelines do not advocate admitting the majority of patients with diabetes mellitus for insulin infusions the night before surgery,^36^ and treating anaemia the day before surgery will not carry additional benefit over treatment at induction of anaesthesia. It was notable that postoperative admission to higher levels of care was also associated with early admission; this finding might support the notion that patients who are considered ‘high risk’ should be admitted early as a ‘precaution’. This might be a cultural phenomenon in some institutions: the unexplained variation between hospital sites (ICC) for day of surgery admission compared with prolonged LOS (52.7% *vs* 6.3%) is a key finding that supports this hypothesis. This requires further investigation, particularly as there is no evidence to support early admission for optimisation which with good planning could not be done as an outpatient, and in fact, patients admitted early might be exposed to greater risk.

Cost-effective solutions, which could have additional benefits, might mitigate the impact of long-distances travelled to hospital from domiciliary residence. This could become increasingly important as more NHS patients exert their constitutional right to choice of treatment location, meaning potentially more patients travelling greater distances in order to receive the care they choose. Furthermore, it is probable that some patients did not travel from their home address, particularly if they lived far from hospital. These patients could have stayed in privately arranged or hospital-arranged accommodation, including hotels. This might have reduced the apparent impact of travel distance in our analysis, but also represents a source of potential inequity; that is, that choice would not be possible for patients who are unable to subsidise the cost of accommodation. Policy makers and hospital providers might wish to consider the opportunity to provide hotel accommodation to patients before surgery to improve equity of access and reduce unnecessary admission before the day of surgery. The cost of an inpatient bed is on average £483,^37^ which is roughly four times that of the average national hotel cost per day of £120.^38^ Providing elective surgical patients with local hotel accommodation and transport to hospital on the day of surgery might be cost-effective, more acceptable to patients, and reduce pressure on inpatient resources. Commercial or medical hotel accommodation options have been implemented in postoperative care, ambulatory cancer treatment, rehabilitation, and isolation of individuals with communicable disease, including in the NHS, and with generally favourable results from the patient and cost perspectives.^39^, ^40^, ^41^, ^42^

This analysis has strengths and limitations. The granularity of the PQIP dataset, and in particular, the level of detail we have about the processes of care that patients underwent, has enabled us to explore potential reasons for admission before the day of surgery which would not be possible using administrative datasets. We were able to control for a comprehensive list of individual health risk factors, factors related to surgical processes, complexity and pathways, and contextual factors such as region of providers, rurality, and socioeconomic deprivation. We conducted our analysis on an individual patient-level basis, and used a mixed-effects model to adjust for clustering of hospital sites, highlighting the potential for hospital-level culture or other factors influencing the likelihood of day of surgery admission.

We also highlight key limitations. Firstly, we have evaluated only a sample of NHS patients, recruited into a research study, and as such there might be some bias in the sampling. Less biased estimates on the number or proportion of NHS patients admitted before the day of surgery might be obtained from administrative data such as Hospital Episode Statistics (HES). However, the @@Model Hospital (model.nhs.uk^43^), which uses HES data to compare day of surgery admission rates between providers, supports our findings: while their denominator is all procedures within a clinical specialty, (including minor or nonsurgical procedures such as injections, cancer therapies or scans) and therefore the proportion of pre-day of surgery admissions is lower, these data still demonstrate substantial variation between NHS providers, and between surgical specialties.

Secondly, we used patient residential LSOA population weighted centroid as a proxy for home address in order to protect the anonymity of participants; however, we contend that the inaccuracy of travel distance or time which results from this approximation is unlikely to affect our overall key findings.

Thirdly, we measured distance to hospital as a crude straight-line distance. Depending on availability of different transport modes and terrain conditions, there might be considerable variation in travel time to hospital even though the straight-line distance is the same. However, we adjusted for rurality of patient location and socioeconomic deprivation, both of which might affect travel time as opposed to distance; other studies have measured travel time and found no significant differences to straight-line distance.^30^

Fourthly, we did not specifically explore the impact of the COVID-19 pandemic that took place during data collection. Only 5% of recruited cases took place during the peak of the pandemic because of both reduced elective surgical volume and reduced national recruitment to research. We are not able to determine whether this impacted local processes for elective surgical admissions.

Finally, as a secondary analysis we were limited to the variables available in the PQIP dataset, so potentially important predictors that might have explained some of the variation observed were not available.

Although the generalisability of these findings might be limited by factors specific to the UK NHS and population geography (for example the UK has comparatively short average travel distances), this work has applicability in an international context as efforts to optimise healthcare efficiency are universal. Previous research has reported efforts to improve day of surgery admission rates across multiple countries and surgical specialties, including for complex major surgery.^44^, ^45^, ^46^, ^47^ Common themes also highlighted in our study include the need for preoperative optimisation and supportive organisational structures and processes.

In conclusion, 17% of patients undergoing elective major surgery recruited into the PQIP study in the UK were admitted in the 24 h before the day of surgery. Increasing distance between home and hospital, inadequate preoperative preparation, and unmeasured local cultures are all risk factors for early hospitalisation which could potentially be explored and addressed through policy and local quality improvement. We suggest that day of surgery admission rates should be monitored with the same focus as other key metrics such as day surgery rates and postoperative LOS. Reasons for early admission should be recorded and analysed at the local level so that providers might explore this opportunity to improve patient experience and safety, reduce hospital occupancy, and improve patient flow through secondary care institutions.

## Authors’ contributions

Conceived the PQIP study and this analysis: SRM

Drafted the first draft and conducted data analysis: BH

Provided guidance, supervised the analysis, and revised the manuscript: TS and SRM

Revised the manuscript and agreed to the final version of the manuscript: all authors

## Funding

The National Institute for Health and Care Research (NIHR) CentralLondon Patient Safety Research Collaboration (CL-PSRC), reference number NIHR204297, and the Royal College of Anaesthetists. BH, JB, DW and AH are supported by the CL-PSRC. SRM and TS are supported by the NIHR University College London Hospitals Biomedical Research Centre (NIHR203328). The views expressed are those of the authors and not necessarily those of the NIHR or the Department of Health and Social Care.

## Declaration of interests

SRM is National Clinical Director for Critical and Perioperative Care and was interim Director of Patient Safety at NHS England during 2025. The other authors declare that they have no conflicts of interest.
