# Supplementary material for: Factors associated with admission before the day of elective major surgery: an analysis of data from the UK Perioperative Quality Improvement Programme
Source: Br J Anaesth. 2025 Dec 31;136(3):948–58. doi: 10.1016/j.bja.2025.10.064 (PMC12975378; doi:10.1016/j.bja.2025.10.064)
Supplement: Multimedia component 1 [file mmc1.docx]

## Supplementary Materials

**S1 Description of distance to hospital**

**Figure S1: Distribution of straight-line distance from home to hospital. The top histogram shows the distribution for the entire study cohort (N=54,267). The middle histogram shows the distribution for the analytical sample used in the primary analysis of pre-day of surgery admission (N=52,213). The bottom histogram shows the distribution for the analytical sample used in the secondary analysis of prolonged postoperative length of stay (N=51,264).**

1. Whole cohort


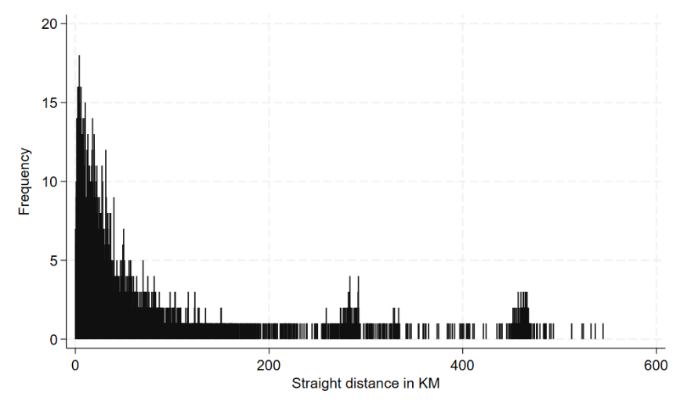


1. Pre-day of surgery admission analysis sample


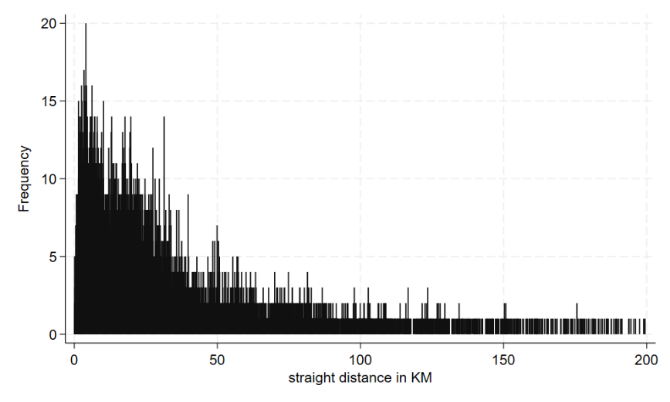


1. Prolonged postoperative length of stay sample


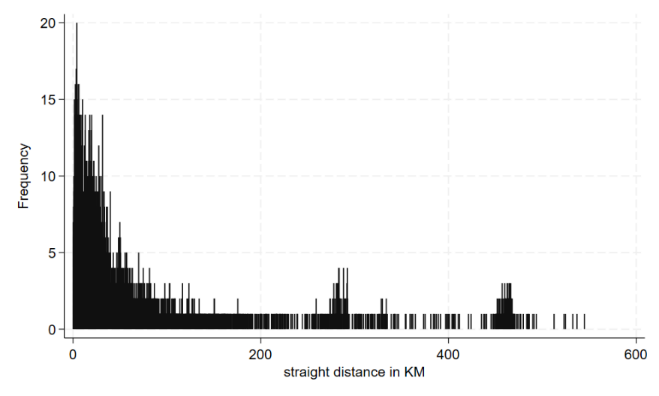


Additional descriptions on distance to hospital were also in Table 1 and Table S4.

**S2 Patients travelled out region for surgery, N=54,267**

**Table S2**

| **Regions** | **In region** | **Out region** | **N** |
| --- | --- | --- | --- |
| North East (England) | 99.34 | 0.66 | 2,122 |
| North West (England) | 97.81 | 2.19 | 7,222 |
| Yorkshire and The Humber | 89.86 | 10.14 | 3,404 |
| East Midlands (England) | 79.66 | 20.34 | 8,939 |
| West Midlands (England) | 95.51 | 4.49 | 4,984 |
| East of England | 70.8 | 29.2 | 2,017 |
| London | 91.21 | 8.79 | 6,744 |
| South East (England) | 74.39 | 25.61 | 9,270 |
| South West (England) | 93.9 | 6.1 | 6,326 |
| Wales | 84.41 | 15.59 | 3,239 |


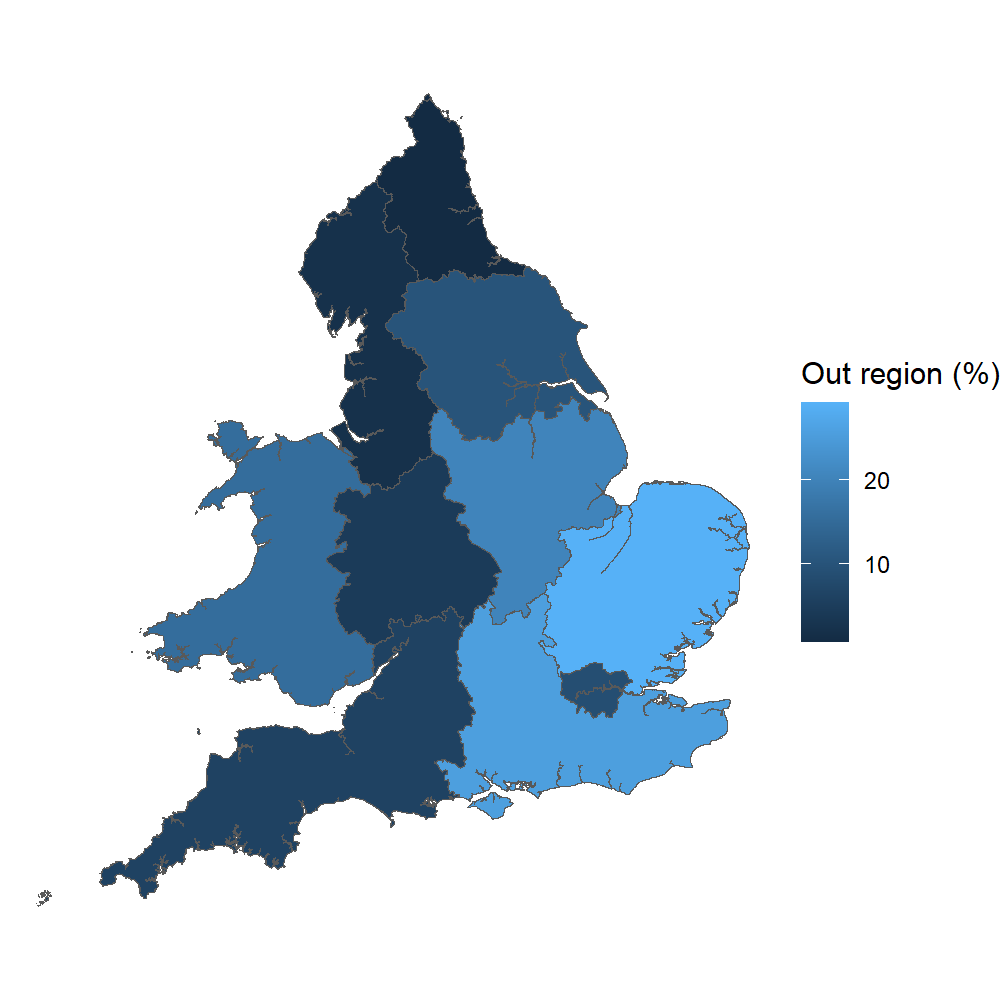


**S3 Adjusted Odds Ratios for Pre-Day of Surgery Admission by Anonymised Hospital**

Figure S3


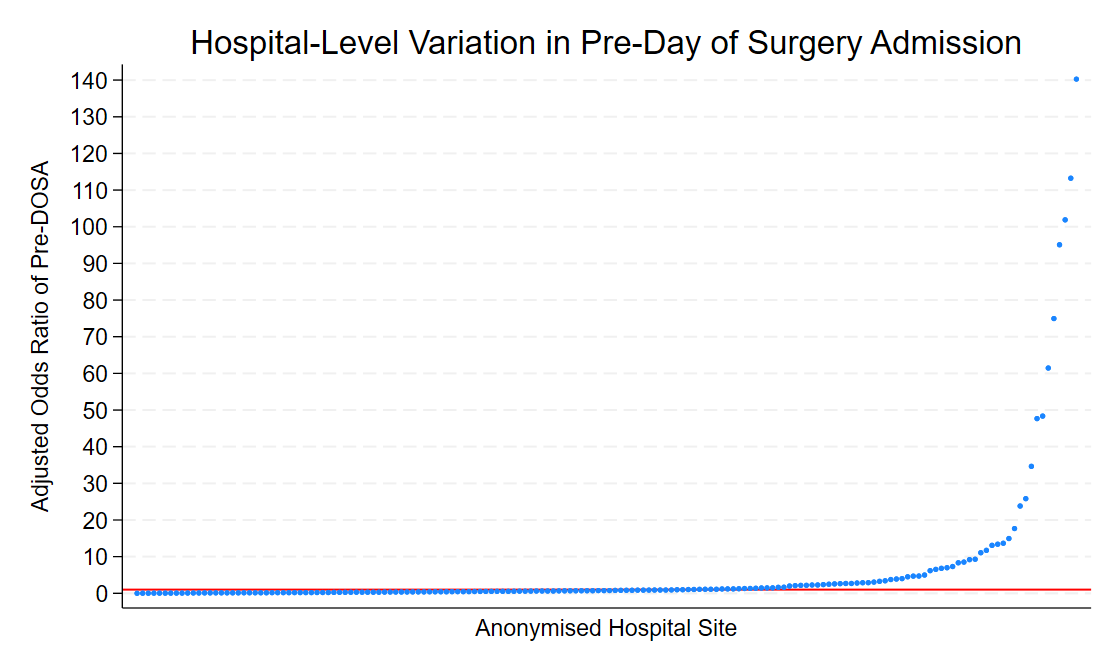


Each dot represents the adjusted odds ratio for an anonymised hospital from the fully adjusted model shown in table 3. The red line indicates an odds ratio of 1.0 and serves as a reference.

**S4 Descriptive statistics by prolonged LOS (0/1), n=51,264**

|  | Prolonged LOS Yes/No | |
| --- | --- | --- |
|  | No (n=46,245) | Yes (n=5,019) |
|  | Median (IQR) | Median (IQR) |
| Straight distance in kilometre | 12.7 (5.8-24.6) | 12.9 (5.9-25.8) |
| Age | 65.0 (56.0-73.0) | 69.0 (59.0-75.0) |
|  | Frequency  % | Frequency  % |

| Gender | No. | Col % | No. | Col % |
| --- | --- | --- | --- | --- |
| F | 20788 | 45 | 2029 | 40.4 |
| M | 25457 | 55 | 2990 | 59.6 |
| Total | 46245 | 100 | 5019 | 100 |
| Cancer diagnosiswithin5years | No. | Col % | No. | Col % |
| No | 15689 | 34 | 1388 | 27.7 |
| Yes | 30498 | 66 | 3625 | 72.3 |
| Total | 46187 | 100 | 5013 | 100 |
| Patient On An Enhanced Recovery Pathway | No. | Col % | No. | Col % |
| Yes | 27114 | 58.6 | 2876 | 57.3 |
| No | 11997 | 25.9 | 1414 | 28.2 |
| Missing | 7134 | 15.4 | 729 | 14.5 |
| Total | 46245 | 100 | 5019 | 100 |
| Preop assessment | No. | Col % | No. | Col % |
| Yes | 45095 | 97.5 | 4810 | 95.8 |
| No | 677 | 1.5 | 120 | 2.4 |
| Missing | 473 | 1 | 89 | 1.8 |
| Total | 46245 | 100 | 5019 | 100 |
| sodium | No. | Col % | No. | Col % |
| Normal | 45160 | 97.7 | 4814 | 95.9 |
| Abnormal | 1085 | 2.3 | 205 | 4.1 |
| Total | 46245 | 100 | 5019 | 100 |
| creatinine | No. | Col % | No. | Col % |
| Normal | 33873 | 73.2 | 3381 | 67.4 |
| Abnormal | 12372 | 26.8 | 1638 | 32.6 |
| Total | 46245 | 100 | 5019 | 100 |
| albumin | No. | Col % | No. | Col % |
| Normal | 43247 | 93.5 | 4433 | 88.3 |
| Abnormal | 2998 | 6.5 | 586 | 11.7 |
| Total | 46245 | 100 | 5019 | 100 |
| White cell | No. | Col % | No. | Col % |
| Normal | 43360 | 93.8 | 4563 | 90.9 |
| Abnormal | 2885 | 6.2 | 456 | 9.1 |
| Total | 46245 | 100 | 5019 | 100 |
| haemoglobin1 | No. | Col % | No. | Col % |
| Normal | 22199 | 48 | 1924 | 38.3 |
| Abnormal | 24046 | 52 | 3095 | 61.7 |
| Total | 46245 | 100 | 5019 | 100 |
| pulserate1 | No. | Col % | No. | Col % |
| Normal | 40506 | 87.6 | 4367 | 87 |
| Abnormal | 5739 | 12.4 | 652 | 13 |
| Total | 46245 | 100 | 5019 | 100 |
| Cerebrovascular Disease | No. | Col % | No. | Col % |
| Yes | 1740 | 3.8 | 256 | 5.1 |
| No | 44448 | 96.2 | 4758 | 94.9 |
| Total | 46188 | 100 | 5014 | 100 |
| Patient has Dementia | No. | Col % | No. | Col % |
| Yes | 277 | 0.6 | 40 | 0.8 |
| No | 45903 | 99.4 | 4973 | 99.2 |
| Total | 46180 | 100 | 5013 | 100 |
| Diabetes | No. | Col % | No. | Col % |
| No diabetes | 40288 | 89.9 | 4147 | 86.6 |
| T1 & HbA1C<=8.5 | 122 | 0.3 | 18 | 0.4 |
| T1 & HbA1C>8.5 | 69 | 0.2 | 12 | 0.3 |
| T2 & HbA1C<=8.5 | 3397 | 7.6 | 471 | 9.8 |
| T2 & HbA1C>8.5 | 936 | 2.1 | 142 | 3 |
| Total | 44812 | 100 | 4790 | 100 |
| ASA grade | No. | Col % | No. | Col % |
| 1&2 | 32571 | 70.6 | 2713 | 54.2 |
| 3-5 | 13576 | 29.4 | 2297 | 45.8 |
| Total | 46147 | 100 | 5010 | 100 |
| NYHA Heart Failure Classification | No. | Col % | No. | Col % |
| I | 38971 | 84.6 | 3910 | 78.1 |
| II | 5995 | 13 | 873 | 17.4 |
| III or IV | 1097 | 2.4 | 221 | 4.4 |
| Total | 46063 | 100 | 5004 | 100 |
| Patient Receive Bowel Preparation | No. | Col % | No. | Col % |
| No/Not known | 34385 | 74.4 | 3559 | 70.9 |
| Yes | 11860 | 25.6 | 1460 | 29.1 |
| Total | 46245 | 100 | 5019 | 100 |
| Rockwood Clinical Frailty Score | No. | Col % | No. | Col % |
| Not frail 1-4 | 27563 | 59.6 | 2705 | 53.9 |
| Mild 5 | 771 | 1.7 | 160 | 3.2 |
| Moderate 6-9 | 432 | 0.9 | 125 | 2.5 |
| Not known | 17479 | 37.8 | 2029 | 40.4 |
| Total | 46245 | 100 | 5019 | 100 |
| BMI | No. | Col % | No. | Col % |
| Low | 514 | 1.1 | 73 | 1.5 |
| Normal/high | 44122 | 95.5 | 4757 | 94.9 |
| Very high | 1546 | 3.3 | 183 | 3.7 |
| Total | 46182 | 100 | 5013 | 100 |
| S02PatientsSmokingHistory | No. | Col % | No. | Col % |
| Current smoker | 4870 | 10.5 | 591 | 11.8 |
| Never smoked | 22445 | 48.5 | 2140 | 42.6 |
| Ex-smoker | 17019 | 36.8 | 2086 | 41.6 |
| Not known | 1911 | 4.1 | 202 | 4 |
| Total | 46245 | 100 | 5019 | 100 |
| S02UrgencyOfSurgery.x | No. | Col % | No. | Col % |
| El | 42403 | 91.7 | 4525 | 90.2 |
| Ex | 3832 | 8.3 | 492 | 9.8 |
| Total | 46235 | 100 | 5017 | 100 |
| duration surgery | No. | Col % | No. | Col % |
| <2 hours | 4509 | 9.9 | 176 | 3.6 |
| 2-3 hours | 11859 | 26 | 695 | 14 |
| >3 hours | 29222 | 64.1 | 4076 | 82.4 |
| Total | 45590 | 100 | 4947 | 100 |
| DrEaMing | No. | Col % | No. | Col % |
| No | 15532 | 33.7 | 3208 | 64.2 |
| Yes | 30570 | 66.3 | 1790 | 35.8 |
| Total | 46102 | 100 | 4998 | 100 |
| Complication | No. | Col % | No. | Col % |
| Grade I and None | 38203 | 82.8 | 1406 | 28.2 |
| Grade II | 6136 | 13.3 | 1652 | 33.2 |
| Grade III and above | 1812 | 3.9 | 1924 | 38.6 |
| Total | 46151 | 100 | 4982 | 100 |
| Pain recovery | No. | Col % | No. | Col % |
| None | 19843 | 43.1 | 1996 | 39.9 |
| Mild | 10215 | 22.2 | 1036 | 20.7 |
| Moderate | 8782 | 19.1 | 914 | 18.3 |
| Severe | 4572 | 9.9 | 513 | 10.3 |
| Unable to ascertain | 2647 | 5.7 | 542 | 10.8 |
| Total | 46059 | 100 | 5001 | 100 |
| Nasogastric Present On Arrival From Theatres | No. | Col % | No. | Col % |
| No | 40246 | 87.3 | 3756 | 75.1 |
| Yes | 5839 | 12.7 | 1246 | 24.9 |
| Total | 46085 | 100 | 5002 | 100 |
| Day of surgery | No. | Col % | No. | Col % |
| Fri | 5294 | 11.4 | 592 | 11.8 |
| Mon | 9201 | 19.9 | 978 | 19.5 |
| Sat | 188 | 0.4 | 18 | 0.4 |
| Sun | 127 | 0.3 | 21 | 0.4 |
| Thu | 9449 | 20.4 | 996 | 19.8 |
| Tue | 11387 | 24.6 | 1187 | 23.7 |
| Wed | 10599 | 22.9 | 1227 | 24.4 |
| Total | 46245 | 100 | 5019 | 100 |
| region sites | No. | Col % | No. | Col % |
| East Midlands | 1321 | 2.9 | 123 | 2.5 |
| East of England | 5925 | 12.8 | 513 | 10.2 |
| London | 8592 | 18.6 | 1375 | 27.4 |
| North East | 2173 | 4.7 | 177 | 3.5 |
| North West | 6320 | 13.7 | 808 | 16.1 |
| South East | 6552 | 14.2 | 566 | 11.3 |
| South West | 5700 | 12.3 | 518 | 10.3 |
| Wales | 2185 | 4.7 | 220 | 4.4 |
| West Midlands | 4687 | 10.1 | 437 | 8.7 |
| Yorkshire and The Humber | 2790 | 6 | 282 | 5.6 |
| Total | 46245 | 100 | 5019 | 100 |
| IMD | No. | Col % | No. | Col % |
| Most deprived quintile | 6031 | 13.8 | 778 | 16.3 |
| 2nd most deprived | 8007 | 18.3 | 944 | 19.8 |
| 3rd most deprived | 9657 | 22.1 | 1019 | 21.4 |
| 2nd least deprived | 9919 | 22.7 | 998 | 20.9 |
| Least deprived quintile | 10030 | 23 | 1026 | 21.5 |
| Total | 43644 | 100 | 4765 | 100 |
| rural/urban | No. | Col % | No. | Col % |
| rural | 10421 | 22.5 | 942 | 18.8 |
| urban | 35824 | 77.5 | 4077 | 81.2 |
| Total | 46245 | 100 | 5019 | 100 |

|  | Prolonged LOS Yes/No | | | |
| --- | --- | --- | --- | --- |
|  | No. | | Yes | |
| Specialty | No. | Col % | No. | Col % |
| Abdominal - Hepatobiliary | 2709 | 5.9 | 292 | 5.8 |
| Abdominal - Lower gastrointestinal | 19410 | 42 | 2076 | 41.4 |
| Abdominal - Upper gastrointestinal | 2716 | 5.9 | 300 | 6 |
| Abdominal - other | 1149 | 2.5 | 130 | 2.6 |
| Burns and Plastics | 1086 | 2.3 | 115 | 2.3 |
| Gynaecology | 2092 | 4.5 | 237 | 4.7 |
| Head and neck | 945 | 2 | 103 | 2.1 |
| Not recorded | 30 | 0.1 | 4 | 0.1 |
| Orthopaedics | 2452 | 5.3 | 256 | 5.1 |
| Spinal | 1105 | 2.4 | 117 | 2.3 |
| Thoracics | 4723 | 10.2 | 520 | 10.4 |
| Urology | 7133 | 15.4 | 791 | 15.8 |
| Vascular | 695 | 1.5 | 78 | 1.6 |
| Total | 46245 | 100 | 5019 | 100 |
| Complexity | No. | Col % | No. | Col % |
| Complex/Complex major | 40764 | 88.1 | 4561 | 90.9 |
| Major/unknown | 5481 | 11.9 | 458 | 9.1 |
| Total | 46245 | 100 | 5019 | 100 |

**S5 Association between distance to hospital, patient and surgical factors, and prolonged LOS.**

Prolonged LOS, mixed effects logistic regression, all patients England and Wales in PQIP, N=48,503, Odds Ratio reported

|  | OR | P value | 95% CI |
| --- | --- | --- | --- |
| Distance to hospital (knot 1 – 1.91 km as reference)a |  | (0.048) |  |
| Knot 2- 6.37 km | 1.1 |  | [0.970, 1.250] |
| Knot 3- 12.76 km | 1.02 |  | [0.900,1.160] |
| Knot 4- 22.82 km | 1.01 |  | [0.890, 1.150] |
| Knot 5- 71.50 km | 1.14 |  | [0.950, 1.370] |
| Centered age | 1.019*** | (<0.001) | [1.015,1.023] |
| Centered age squared | 1.000*** | (<0.001) | [1.000,1.000] |
| Male sex | 1.167** | (0.001) | [1.061,1.283] |
| Ref: Abdominal-Lower GI, Complex /complex major |  |  |  |
| Abdominal-H,Complex/complex maj | 0.362*** | (<0.001) | [0.254,0.515] |
| Abdominal-H,major/not known | 0.28 | (0.117) | [0.057,1.373] |
| Abdominal-Lower GI,major/not known | 0.618 | (0.111) | [0.342,1.116] |
| Abdominal-Upper GI,Complex/complex maj | 0.179*** | (<0.001) | [0.128,0.250] |
| Abdominal-Upper GI,major/not known | 0.098* | (0.014) | [0.015,0.631] |
| Abdominal-Other,Complex/complex maj | 0.699 | (0.074) | [0.472,1.035] |
| Abdominal-Other,major/not known | 0.132** | (0.003) | [0.034,0.506] |
| Burns and Plastics,Complex/complex maj | 7.856*** | (<0.001) | [3.008,20.518] |
| Gynaecology,Complex/complex maj | 4.074*** | (<0.001) | [2.731,6.079] |
| Head and neck,Complex/complex maj | 0.410*** | (<0.001) | [0.252,0.665] |
| Head and neck,major/not known | 0.086 | (0.202) | [0.002,3.738] |
| Orthopaedics,Complex/complex maj | 1.424 | (0.065) | [0.979,2.072] |
| Spinal,Complex/complex maj | 1.988 | (0.061) | [0.968,4.083] |
| Thoracics,Complex/complex maj | 2.996*** | (0.001) | [2.255,3.979] |
| Thoracics,major/not known | 2.337*** | (0.001) | [1.867,2.925] |
| Urology,Complex/complex maj | 2.478*** | (0.001) | [1.973,3.113] |
| Urology,major/not known | 1.05 | (0.732) | [0.794,1.389] |
| Vascular,Complex/complex maj | 0.901 | (0.575) | [0.627,1.295] |
| Cancer diagnosis or in remission for <5 years : ref No |  |  |  |
| Yes | 1.002 | (0.969) | [0.902,1.114] |
| Enhanced recovery: ref Yes |  |  |  |
| No | 1.058 | (0.506) | [0.896,1.250] |
| Unknown | 1.114 | (0.149) | [0.962,1.291] |
| Preop-assessment: ref Yes |  |  |  |
| No | 1.738*** | (<0.001) | [1.348,2.241] |
| Unknown | 0.881 | (0.616) | [0.537,1.445] |
| Sodium: ref Normal |  |  |  |
| Abnormal | 1.252* | (0.028) | [1.025,1.528] |
| Creatinine: ref Normal |  |  |  |
| Abnormal | 1.07 | (0.102) | [0.987,1.160] |
| Albumin: ref Normal |  |  |  |
| Abnormal | 1.403*** | (<0.001) | [1.236,1.593] |
| White cell count:ref Normal |  |  |  |
| Abnormal | 1.152 | (0.051) | [0.999,1.328] |
| Haemoglobin: ref Normal |  |  |  |
| Abnormal | 1.183*** | (0.001) | [1.074,1.303] |
| Pulse rate: ref Normal |  |  |  |
| Abnormal | 0.963 | (0.529) | [0.858,1.082] |
| Cerebrovascular Disease: No |  |  |  |
| Yes | 1.006 | (0.949) | [0.845,1.196] |
| Dementia: No |  |  |  |
| Yes | 1.459 | (0.098) | [0.933,2.284] |
| Diabetes: ref No diabetes |  |  |  |
| T1 & HbA1C<=8.5 | 1.194 | (0.574) | [0.643,2.215] |
| T1 & HbA1C>8.5 | 1.523 | (0.290) | [0.698,3.319] |
| T2 & HbA1C<=8.5 | 0.985 | (0.813) | [0.870,1.115] |
| T2 & HbA1C>8.5 | 1.246* | (0.023) | [1.030,1.506] |
| ASA grade: ref 1&2 |  |  |  |
| 3-5 | 1.387*** | (<0.001) | [1.251,1.538] |
| NYHA: ref class I |  |  |  |
| II | 1.068 | (0.228) | [0.960,1.188] |
| III or IV | 1.056 | (0.637) | [0.843,1.323] |
| Bowel prep: ref No/Not known |  |  |  |
| Yes | 1.203** | (0.002) | [1.068,1.354] |
| Frailty: ref Not frail 1-4 |  |  |  |
| Mild 5 | 1.445** | (0.001) | [1.152,1.813] |
| Moderate 6-9 | 2.105*** | (<0.001) | [1.632,2.716] |
| Not done or not known | 1.135* | (0.044) | [1.003,1.285] |
| BMI: Normal/high |  |  |  |
| Low | 1.188 | (0.280) | [0.870,1.622] |
| Very high | 1.009 | (0.924) | [0.834,1.222] |
| Smoking: ref never smoked |  |  |  |
| Current smoker | 1.131 | (0.061) | [0.994,1.287] |
| Ex-smoker | 1.061 | (0.172) | [0.975,1.155] |
| Not known | 1.094 | (0.481) | [0.852,1.404] |
| Urgency of Surgery: ref El |  |  |  |
| Ex | 1.028 | (0.692) | [0.898,1.176] |
| Duration of surgery: ref <2 hours |  |  |  |
| 2-3 hours | 1.518*** | (<0.001) | [1.288,1.789] |
| >3 hours | 2.770*** | (<0.001) | [2.285,3.358] |
| DrEaMing: ref No |  |  |  |
| Yes | 0.420*** | (<0.001) | [0.363,0.486] |
| Post-op complications: ref Grade I |  |  |  |
| Grade II | 6.714*** | (<0.001) | [5.900,7.642] |
| Grade III and above | 35.081*** | (<0.001) | [29.334,41.953] |
| Pain recovery: ref None |  |  |  |
| Mild | 0.969 | (0.416) | [0.899,1.045] |
| Moderate | 1.053 | (0.381) | [0.938,1.182] |
| Severe | 0.952 | (0.478) | [0.829,1.092] |
| Unable to ascertain | 1.330** | (0.002) | [1.108,1.597] |
| Nasogastric tube postop: ref No |  |  |  |
| Yes | 1.727*** | (0.001) | [1.480,2.015] |
| Day of surgery: ref Tuesday |  |  |  |
| Fri | 1.047 | (0.565) | [0.895,1.226] |
| Mon | 1.09 | (0.165) | [0.965,1.231] |
| Sat | 0.964 | (0.911) | [0.511,1.821] |
| Sun | 2.200** | (0.003) | [1.297,3.731] |
| Thu | 0.965 | (0.546) | [0.858,1.084] |
| Wed | 1.134 | (0.122) | [0.967,1.330] |
| Provider region: East of England |  |  |  |
| London | 1.334 | (0.116) | [0.931,1.912] |
| North East | 0.912 | (0.759) | [0.506,1.644] |
| North West | 1.622** | (0.004) | [1.162,2.262] |
| South East | 1.038 | (0.852) | [0.699,1.542] |
| South West | 1.211 | (0.305) | [0.840,1.747] |
| Wales | 1.209 | (0.295) | [0.847,1.726] |
| East Midlands | 1.518* | (0.037) | [1.026,2.248] |
| West Midlands | 1.23 | (0.328) | [0.812,1.861] |
| Yorkshire and The Humber | 1.172 | (0.491) | [0.746,1.843] |
| Rurality: ref rural |  |  |  |
| urban | 1.151* | (0.010) | [1.034,1.282] |
| Observations | 48503 |  |  |
| Intraclass correlation coefficient | 0.063 |  |  |

^*^ *p* < 0.05, ^**^ *p* < 0.01, ^***^ *p* < 0.001

**S6 Main analyses additionally adjusted for 2019 IMD quintiles**

**Table S6a Distance to Hospital and Admission before Day of Surgery including 2019 IMD- patients from England only, mixed effects logistic regression, OR reported (N=45766), Odds Ratio reported**

|  | OR | P value | 95% CI |
| --- | --- | --- | --- |
| Distance to hospital (knot 1 – 1.91 km as reference)^a^ |  | (<0.001) |  |
| Knot 2- 6.36 km | 1.000 |  | [0.880,1.140] |
| Knot 3- 12.71 km | 1.090 |  | [0.920,1.300] |
| Knot 4- 22.30 km | 1.310 |  | [1.110,1.540] |
| Knot 5- 62.57 km | 1.910 |  | [1.610,2.260] |
| centered age | 1.008** | (0.003) | [1.003,1.014] |
| centered age squared | 1.000*** | (<0.001) | [1.000,1.000] |
| Male sex | 1.099 | (0.094) | [0.984,1.226] |
| Ref: Abdominal-Lower GI, Complex /complex major |  |  |  |
| Abdominal-H,Complex/complex maj | 2.792** | (0.008) | [1.312,5.940] |
| Abdominal-H,major/not known | 2.63 | (0.157) | [0.689,10.037] |
| Abdominal-Lower GI,major/not known | 1.607 | (0.126) | [0.875,2.954] |
| Abdominal-Upper GI,Complex/complex maj | 3.894* | (0.046) | [1.023,14.825] |
| Abdominal-Upper GI,major/not known | 2.051* | (0.037) | [1.046,4.020] |
| Abdominal-Other,Complex/complex maj | 1.326 | (0.287) | [0.789,2.227] |
| Abdominal-Other,major/not known | 1.154 | (0.798) | [0.385,3.460] |
| Burns and Plastics,Complex/complex maj | 3.224 | (0.320) | [0.321,32.421] |
| Gynaecology,Complex/complex maj | 0.653 | (0.373) | [0.255,1.669] |
| Gynaecology,major/not known | 1.03 | (0.985) | [0.049,21.667] |
| Head and neck,Complex/complex maj | 17.991*** | (<0.001) | [6.761,47.871] |
| Head and neck,major/not known | 61.267** | (0.006) | [3.213,1168.431] |
| Orthopaedics,Complex/complex maj | 0.455 | (0.131) | [0.164,1.262] |
| Spinal,Complex/complex maj | 4.879*** | (<0.001) | [2.036,11.691] |
| Spinal,major/not known | 75.314*** | (<0.001) | [12.300,461.139] |
| Thoracics,Complex/complex maj | 11.524*** | (<0.001) | [4.130,32.154] |
| Thoracics,major/not known | 13.812*** | (<0.001) | [4.751,40.153] |
| Urology,Complex/complex maj | 1.375 | (0.480) | [0.569,3.321] |
| Urology,major/not known | 1.009 | (0.982) | [0.462,2.205] |
| Vascular,Complex/complex maj | 3.434* | (0.020) | [1.218,9.681] |
| Urgency of Surgery: ref Elective |  |  |  |
| Expedited | 1.279 | (0.124) | [0.935,1.751] |
| Cancer diagnosis or in remission for <5 years: ref No |  |  |  |
| Yes | 1.185 | (0.162) | [0.934,1.504] |
| Enhanced recovery: ref Yes |  |  |  |
| No | 0.954 | (0.781) | [0.683,1.332] |
| Unknown | 0.883 | (0.268) | [0.709,1.100] |
| Preop-assessment: ref Yes |  |  |  |
| No | 1.927 | (0.147) | [0.795,4.673] |
| Sodium: ref Normal |  |  |  |
| Abnormal | 1.069 | (0.578) | [0.844,1.355] |
| Creatinine: ref Normal |  |  |  |
| Abnormal | 1.141** | (0.001) | [1.052,1.237] |
| Albumin: ref Normal |  |  |  |
| Abnormal | 1.363*** | (<0.001) | [1.173,1.583] |
| White cell count:ref Normal |  |  |  |
| Abnormal | 1.048 | (0.634) | [0.865,1.270] |
| Haemoglobin: ref Normal |  |  |  |
| Abnormal | 1.363*** | (<0.001) | [1.235,1.505] |
| Pulse rate: ref Normal |  |  |  |
| Abnormal | 0.972 | (0.628) | [0.866,1.091] |
| Cerebrovascular Disease: No |  |  |  |
| Yes | 1.101 | (0.314) | [0.913,1.329] |
| Dementia: No |  |  |  |
| Yes | 0.817 | (0.311) | [0.553,1.208] |
| Diabetes: ref No diabetes |  |  |  |
| T1 & HbA1C<=8.5 | 2.383* | (0.027) | [1.104,5.141] |
| T1 & HbA1C>8.5 | 2.970** | (0.003) | [1.447,6.098] |
| T2 & HbA1C<=8.5 | 1.045 | (0.539) | [0.907,1.205] |
| T2 & HbA1C>8.5 | 1.456** | (0.002) | [1.152,1.840] |
| ASA grade: ref 1&2 |  |  |  |
| 3-5 | 1.196* | (0.020) | [1.028,1.391] |
| Planned post-op destination: ref Ward care |  |  |  |
| Level 1 care | 1.111 | (0.518) | [0.807,1.531] |
| Level 2 care | 2.546*** | (<0.001) | [1.841,3.521] |
| Level 3 care | 3.866*** | (<0.001) | [2.150,6.954] |
| Bowel prep: ref No |  |  |  |
| Yes | 2.260*** | (<0.001) | [1.435,3.561] |
| Frailty: ref Not frail 1-4 |  |  |  |
| Mild 5 | 1.382* | (0.042) | [1.012,1.886] |
| Moderate 6-9 | 1.882** | (0.002) | [1.264,2.803] |
| Not done or not known | 1.475* | (0.010) | [1.095,1.986] |
| BMI: Normal/high |  |  |  |
| Low | 1.3 | (0.162) | [0.900,1.878] |
| Very high | 0.805 | (0.051) | [0.648,1.001] |
| Day of Surgery: ref Tue |  |  |  |
| Fri | 0.828 | (0.271) | [0.592,1.159] |
| Mon | 0.681** | (0.003) | [0.528,0.878] |
| Sat | 1.521 | (0.507) | [0.440,5.252] |
| Sun | 0.49 | (0.222) | [0.156,1.538] |
| Thu | 1.136 | (0.368) | [0.860,1.501] |
| Wed | 1.117 | (0.346) | [0.888,1.405] |
| Provider region: ref East of England |  |  |  |
| East Midlands | 0.518 | (0.519) | [0.070,3.829] |
| London | 2.813* | (0.039) | [1.056,7.493] |
| North East | 2.106 | (0.268) | [0.564,7.863] |
| North West | 8.405*** | (<0.001) | [3.010,23.469] |
| South East | 1.136 | (0.781) | [0.461,2.799] |
| South West | 0.631 | (0.357) | [0.237,1.681] |
| Wales | 2.5 | (0.650) | [0.048,130.807] |
| West Midlands | 1.542 | (0.554) | [0.367,6.482] |
| Yorkshire and The Humber | 2.839 | (0.150) | [0.687,11.737] |
| IMD 2019: ref least deprived quintile |  |  |  |
| Most deprived quintile | 1.023 | (0.706) | [0.909,1.151] |
| 2nd most deprived | 0.996 | (0.934) | [0.895,1.108] |
| 3rd most deprived | 1.008 | (0.889) | [0.900,1.129] |
| 2nd least deprived | 0.998 | (0.968) | [0.894,1.114] |
| Rurality: ref rural |  |  |  |
| urban | 1.041 | (0.473) | [0.933,1.162] |
| Observations | 45766 |  |  |
| Intraclass correlation coefficient | 0.499 |  |  |

^*^ *p* < 0.05, ^**^ *p* < 0.01, ^***^ *p* < 0.001

**Table S6b Prolonged LOS, 2019 IMD included - patients from England only, mixed effects logistic regression, Odds Ratio reported, N=45,790**

|  | OR | P value | 95% CI |
| --- | --- | --- | --- |
| Distance to hospital (knot 1 – 1.91 km as reference)^a^ |  | 0.054 |  |
| Knot 2- 6.37 km | 1.12 |  | [0.980,1.270] |
| Knot 3- 12.76 km | 1.04 |  | [0.920,1.190] |
| Knot 4- 22.82 km | 1.03 |  | [0.900,1.170] |
| Knot 5- 71.50 km | 1.17 |  | [0.960,1.420] |
| Centered age | 1.019^***^ | (<0.001) | [1.015,1.023] |
| Centered age2 | 1.000^***^ | (0.001) | [1.000,1.000] |
| Male sex | 1.155^**^ | (0.004) | [1.048,1.273] |
| Ref: Abdominal-Lower GI, Complex /complex major |  |  |  |
| Abdominal-H,Complex/complex maj | 0.364^***^ | (<0.001) | [0.253,0.524] |
| Abdominal-H,major/not known | 0.275 | (0.111) | [0.056,1.347] |
| Abdominal-Lower GI,major/not known | 0.611 | (0.119) | [0.329,1.135] |
| Abdominal-Upper GI,Complex/complex maj | 0.177^***^ | (<0.001) | [0.125,0.250] |
| Abdominal-Upper GI,major/not known | 0.099^*^ | (0.014) | [0.016,0.630] |
| Abdominal-Other,Complex/complex maj | 0.675^*^ | (0.045) | [0.459,0.992] |
| Abdominal-Other,major/not known | 0.131^**^ | (0.004) | [0.034,0.514] |
| Burns and Plastics,Complex/complex maj | 8.022^***^ | (<0.001) | [3.020,21.306] |
| Gynaecology,Complex/complex maj | 4.164^***^ | (<0.001) | [2.660,6.518] |
| Head and neck,Complex/complex maj | 0.402^***^ | (<0.001) | [0.246,0.657] |
| Head and neck,major/not known | 0.087 | (0.194) | [0.002,3.448] |
| Orthopaedics,Complex/complex maj | 1.321 | (0.158) | [0.898,1.942] |
| Spinal,Complex/complex maj | 2.043^*^ | (0.039) | [1.038,4.021] |
| Thoracics,Complex/complex maj | 2.862^***^ | (<0.001) | [2.136,3.835] |
| Thoracics,major/not known | 2.268^***^ | (<0.001) | [1.797,2.862] |
| Urology,Complex/complex maj | 2.444^***^ | (<0.001) | [1.917,3.117] |
| Urology,major/not known | 1.01 | (0.945) | [0.756,1.350] |
| Vascular,Complex/complex maj | 0.872 | (0.483) | [0.594,1.280] |
| Cancer diagnosis or in remission for <5 years : ref |  |  |  |
| Yes | 0.991 | (0.864) | [0.889,1.103] |
| Enhanced recovery: ref Yes |  |  |  |
| No | 1.04 | (0.660) | [0.873,1.240] |
| Unknown | 1.115 | (0.163) | [0.957,1.301] |
| Preop-assessment: ref Yes |  |  |  |
| No | 1.686^***^ | (<0.001) | [1.279,2.223] |
| Unknown | 0.854 | (0.508) | [0.535,1.364] |
| Sodium: ref Normal |  |  |  |
| Abnormal | 1.261^*^ | (0.027) | [1.026,1.551] |
| Creatinine: ref Normal |  |  |  |
| Abnormal | 1.07 | (0.112) | [0.984,1.162] |
| Albumin: ref Normal |  |  |  |
| Abnormal | 1.409^***^ | (<0.001) | [1.236,1.606] |
| White cell count:ref Normal |  |  |  |
| Abnormal | 1.141 | (0.075) | [0.987,1.319] |
| Haemoglobin: ref Normal |  |  |  |
| Abnormal | 1.168^**^ | (0.003) | [1.054,1.294] |
| Pulse rate: ref Normal |  |  |  |
| Abnormal | 0.991 | (0.879) | [0.879,1.116] |
| Cerebrovascular Disease: No |  |  |  |
| Yes | 1.006 | (0.945) | [0.837,1.211] |
| Dementia: No |  |  |  |
| Yes | 1.515 | (0.066) | [0.972,2.360] |
| Diabetes: ref No diabetes |  |  |  |
| T1 & HbA1C<=8.5 | 1.273 | (0.449) | [0.682,2.374] |
| T1 & HbA1C>8.5 | 1.475 | (0.337) | [0.667,3.262] |
| T2 & HbA1C<=8.5 | 0.956 | (0.481) | [0.843,1.084] |
| T2 & HbA1C>8.5 | 1.223^*^ | (0.045) | [1.004,1.488] |
| ASA grade: ref 1&2 |  |  |  |
| 3-5 | 1.379^***^ | (<0.001) | [1.241,1.531] |
| NYHA: ref class I |  |  |  |
| II | 1.083 | (0.150) | [0.971,1.208] |
| III or IV | 1.084 | (0.489) | [0.863,1.363] |
| Bowel prep: ref No/Not known |  |  |  |
| Yes | 1.208^**^ | (0.003) | [1.065,1.370] |
| Frailty: ref Not frail 1-4 |  |  |  |
| Mild 5 | 1.402^**^ | (0.006) | [1.103,1.783] |
| Moderate 6-9 | 2.161^***^ | (<0.001) | [1.651,2.827] |
| Not done or not known | 1.148^*^ | (0.034) | [1.011,1.304] |
| BMI: Normal/high |  |  |  |
| Low | 1.201 | (0.257) | [0.875,1.648] |
| Very high | 0.969 | (0.756) | [0.794,1.182] |
| Never smoked as ref |  |  |  |
| Current smoker | 1.125 | (0.086) | [0.984,1.287] |
| Ex-smoker | 1.063 | (0.160) | [0.976,1.158] |
| Not known | 1.094 | (0.500) | [0.843,1.419] |
| Urgency of Surgery: ref El |  |  |  |
| Ex | 1.023 | (0.765) | [0.882,1.187] |
| Duration of surgery: ref <2 hours |  |  |  |
| 2-3 hours | 1.469^***^ | (<0.001) | [1.238,1.743] |
| >3 hours | 2.662^***^ | (<0.001) | [2.179,3.251] |
| DrEaMing: ref No |  |  |  |
| Yes | 0.420^***^ | (<0.001) | [0.360,0.489] |
| Post-op complications: ref Grade I |  |  |  |
| Grade II | 6.663^***^ | (<0.001) | [5.831,7.613] |
| Grade III and above | 33.978^***^ | (<0.001) | [28.439,40.596] |
| Pain recovery: ref None |  |  |  |
| Mild | 0.976 | (0.537) | [0.902,1.055] |
| Moderate | 1.069 | (0.270) | [0.950,1.203] |
| Severe | 0.959 | (0.534) | [0.841,1.094] |
| Unable to ascertain | 1.352^**^ | (0.002) | [1.120,1.632] |
| Nasogastric tube postop: ref No |  |  |  |
| Yes | 1.725^***^ | (<0.001) | [1.475,2.018] |
| Day of surgery: ref Tuesday |  |  |  |
| Fri | 1.047 | (0.582) | [0.890,1.231] |
| Mon | 1.102 | (0.140) | [0.969,1.254] |
| Sat | 1.033 | (0.918) | [0.554,1.926] |
| Sun | 2.203^**^ | (0.003) | [1.304,3.723] |
| Thu | 0.943 | (0.341) | [0.835,1.064] |
| Wed | 1.124 | (0.174) | [0.950,1.329] |
| Provider region: East of England |  |  |  |
| East Midlands | 1.467 | (0.062) | [0.982,2.191] |
| London | 1.305 | (0.161) | [0.899,1.892] |
| North East | 0.872 | (0.650) | [0.481,1.578] |
| North West | 1.548^*^ | (0.013) | [1.096,2.186] |
| South East | 1.023 | (0.912) | [0.683,1.531] |
| South West | 1.162 | (0.441) | [0.793,1.703] |
| West Midlands | 1.163 | (0.489) | [0.758,1.785] |
| Yorkshire and The Humber | 1.133 | (0.596) | [0.715,1.794] |
| Rurality: ref rural |  |  |  |
| urban | 1.137^*^ | (0.027) | [1.015,1.273] |
| Least deprived quintile |  |  |  |
| Most deprived quintile | 1.138* | (0.020) | [1.021,1.267] |
| 2nd most deprived | 1.072 | (0.248) | [0.953,1.206] |
| 3rd most deprived | 1.022 | (0.663) | [0.928,1.124] |
| 2nd least deprived | 0.973 | (0.611) | [0.875,1.081] |
| Observations | 45790 |  |  |
| Intraclass correlation coefficient | 0.066 |  |  |

^*^ *p* < 0.05, ^**^ *p* < 0.01, ^***^ *p* < 0.001

**S7 Sensitivity tests of using different forms of distance to hospital**

S7a Sensitivity tests on Day of Surgery Admission (DOSA) and distance to hospital

| Model 1 – distance as a continuous variable in fully adjusted model | Outcome  DOSA – Yes 0/No 1, mixed logistic regression, fully adjusted model | Model 2 – distance as a categorical variable with 3 categories in fully adjusted model | Outcome DOSA (Yes 0/No 1)  mixed logistic regression, fully adjusted model |
| --- | --- | --- | --- |
|  | OR [95 CI] |  | OR [95 CI] |
| Straight distance | 1.009*** [1.007,1.012] | <10 km as ref | 1.000 [1.000,1.000] |
|  |  | 10-30 km | 1.198** [1.064,1.349] |
|  |  | > 30km | 1.817*** [1.589,2.078] |
| Observations | 48369 | Observations | 48369 |

^*^ *p* < 0.05, ^**^ *p* < 0.01, ^***^ *p* < 0.001

S7b Sensitivity tests on prolonged post-surgery LOS and distance to hospital

| Model 1 – distance as a continuous variable in fully adjusted model | Outcome  Prolonged LOS (Yes/No), mixed logistic regression, fully adjusted model | Model 2 – distance as a categorical variable with 3 categories in fully adjusted model | Outcome  Prolonged LOS (Yes/No)  mixed logistic regression, fully adjusted model |
| --- | --- | --- | --- |
|  | OR [95 CI] |  | OR [95 CI] |
| Straight distance | 1.001* | <10 km as ref | 1.000 [1.000,1.000] |
|  | [1.000,1.002] | 10-30 km | 0.940 [0.862,1.024] |
|  |  | > 30km | 1.061 [0.921,1.220] |
| Observations | 48503 |  | 48503 |

^*^ *p* < 0.05, ^**^ *p* < 0.01, ^***^ *p* < 0.001

**S8 Missing data and multiple Imputation by chained equations**

For both primary and secondary analysis, there were no missing data in our outcome and key variable of interest- distance from home to hospital. There were small amount of missing data in our confounders- less than 5%. Variables with more than 10% missing were frailty and enhanced recovery pathway, and in white cell count and serum albumin test. For main analyses, prior to applying complete case analysis, missing values for white cell count and serum albumin were categorized as normal.

To assess the impact of missing data in confounders, multiple imputation by chained equations (MICE) was conducted using Stata 18. This was performed separately for the primary and secondary outcome models, given their different analytical samples. Fifteen imputations were generated, each with 10 iterations. Continuous serum variables were winsorized (1st and 99th percentiles) and then imputed using linear regression. Binary, ordinal, and categorical variables were imputed using logistic, ordered logistic, and multinomial logistic regression, respectively. Predictive mean matching was employed for any variables that failed to converge during the imputation process.

The mixed-effects logistic regression models were subsequently run on the imputed datasets. The xbrcspline Stata package, used to model distance with restricted cubic splines in the primary analysis, is not compatible with imputed datasets. Therefore, an alternative specification for the straight-line distance variable was used in these MI-based sensitivity analyses, e.g., distance as a categorical variable as shown in S8a.

The main study conclusions remained robust when tested with these multiple imputation sensitivity analyses. Detailed results tables from the imputed datasets are provided below.

Table S8a Distance to Hospital and DOSA on imputed dataset, mixed effects logistic regression, OR reported (N=52,199), Odds Ratio reported

|  | OR | P value | 95 CI | |
| --- | --- | --- | --- | --- |
| Straight_distance: ref <10km |  |  |  | |
| 10-30 km | 1.229 | 0.001 | 1.088 | 1.389 |
| > 30km | 1.849 | <0.001 | 1.635 | 2.091 |
|  |  |  |  |  |
| centered_age | 1.008 | 0.007 | 1.002 | 1.013 |
| centered_age2 | 1.000 | 0.001 | 1.000 | 1.000 |
|  |  |  |  |  |
| gender |  |  |  |  |
| Male sex | 1.122 | 0.004 | 1.039 | 1.212 |
|  |  |  |  |  |
| complex_specialty |  |  |  |  |
| Abdominal-H,Complex/complex maj | 2.169 | 0.060 | 0.968 | 4.859 |
| Abdominal-H,major/not known | 1.596 | 0.510 | 0.397 | 6.414 |
| Abdominal-Lower GI,major/not known | 1.557 | 0.096 | 0.924 | 2.623 |
| Abdominal-Upper GI,Complex/complex maj | 2.989 | 0.071 | 0.912 | 9.794 |
| Abdominal-Upper GI,major/not known | 1.820 | 0.079 | 0.932 | 3.553 |
| Abdominal-Other,Complex/complex maj | 1.344 | 0.233 | 0.826 | 2.186 |
| Abdominal-Other,major/not known | 1.005 | 0.993 | 0.336 | 3.004 |
| Burns and Plastics,Complex/complex maj | 1.729 | 0.652 | 0.160 | 18.735 |
| Gynaecology,Complex/complex maj | 0.660 | 0.501 | 0.197 | 2.215 |
| Gynaecology,major/not known | 1.242 | 0.844 | 0.144 | 10.717 |
| Head and neck,Complex/complex maj | 11.228 | <0.001 | 4.160 | 30.306 |
| Head and neck,major/not known | 28.524 | 0.008 | 2.397 | 339.368 |
| Orthopaedics,Complex/complex maj | 0.482 | 0.037 | 0.242 | 0.958 |
| Spinal,Complex/complex maj | 3.667 | 0.005 | 1.470 | 9.146 |
| Spinal,major/not known | 39.900 | <0.001 | 7.038 | 226.187 |
| Thoracics,Complex/complex maj | 6.607 | <0.001 | 2.464 | 17.719 |
| Thoracics,major/not known | 7.568 | <0.001 | 2.621 | 21.852 |
| Urology,Complex/complex maj | 1.071 | 0.861 | 0.499 | 2.298 |
| Urology,major/not known | 0.923 | 0.826 | 0.452 | 1.884 |
| Vascular,Complex/complex maj | 2.411 | 0.075 | 0.915 | 6.349 |
| Not recorded,major/not known | 1.411 | 0.412 | 0.620 | 3.215 |
|  |  |  |  |  |
| Urgency of Surgery: ref Elective |  |  |  |  |
| Expedited | 1.371 | 0.021 | 1.049 | 1.792 |
|  |  |  |  |  |
| cancer_diagnosiswithin5years: ref no |  |  |  |  |
| Yes | 1.273 | 0.024 | 1.033 | 1.568 |
|  |  |  |  |  |
| Enhanced recovery: ref Yes |  |  |  |  |
| No | 1.009 | 0.944 | 0.783 | 1.301 |
|  |  |  |  |  |
| Preop-assessment: ref Yes |  |  |  |  |
| No | 2.029 | 0.059 | 0.973 | 4.230 |
|  |  |  |  |  |
| Sodium: ref Normal |  |  |  |  |
| Abnormal | 1.155 | 0.245 | 0.906 | 1.471 |
|  |  |  |  |  |
| Creatinine: ref Normal |  |  |  |  |
| Abnormal | 1.121 | 0.001 | 1.048 | 1.199 |
|  |  |  |  |  |
| Albumin: ref Normal |  |  |  |  |
| Abnormal | 1.115 | 0.206 | 0.942 | 1.319 |
|  |  |  |  |  |
| White cell count:ref Normal |  |  |  |  |
| Abnormal | 0.919 | 0.256 | 0.793 | 1.064 |
|  |  |  |  |  |
| Haemoglobin: ref Normal |  |  |  |  |
| Abnormal | 1.309 | 0.000 | 1.188 | 1.443 |
|  |  |  |  |  |
| Pulse rate: ref Normal |  |  |  |  |
| Abnormal | 0.982 | 0.732 | 0.887 | 1.088 |
|  |  |  |  |  |
| Cerebrovascular Disease: No |  |  |  |  |
| Yes | 1.035 | 0.701 | 0.869 | 1.232 |
|  |  |  |  |  |
| Dementia: ref No |  |  |  |  |
| Yes | 0.737 | 0.076 | 0.525 | 1.033 |
|  |  |  |  |  |
| Diabetes: ref No diabetes |  |  |  |  |
| T1 & HbA1C<=8.5 | 2.396 | 0.012 | 1.211 | 4.738 |
| T1 & HbA1C>8.5 | 2.309 | 0.031 | 1.079 | 4.945 |
| T2 & HbA1C<=8.5 | 1.051 | 0.454 | 0.922 | 1.199 |
| T2 & HbA1C>8.5 | 1.372 | 0.005 | 1.102 | 1.707 |
|  |  |  |  |  |
| ASA grade: ref 1&2 |  |  |  |  |
| 3-5 | 1.152 | 0.035 | 1.010 | 1.315 |
|  |  |  |  |  |
| Planned post-op destination: ref Ward care |  |  |  |  |
| Level 1 care | 1.301 | 0.112 | 0.940 | 1.801 |
| Level 2 care | 2.681 | <0.001 | 1.989 | 3.612 |
| Level 3 care | 4.076 | <0.001 | 2.395 | 6.937 |
|  |  |  |  |  |
| Bowel prep: ref No |  |  |  |  |
| Yes | 1.580 | 0.003 | 1.170 | 2.133 |
|  |  |  |  |  |
| Frailty: ref Not frail 1-4 |  |  |  |  |
| Mild 5 | 1.200 | 0.166 | 0.927 | 1.553 |
| Moderate 6-9 | 1.494 | 0.008 | 1.110 | 2.009 |
|  |  |  |  |  |
| BMI: Normal/high |  |  |  |  |
| Low | 1.366 | 0.034 | 1.025 | 1.822 |
| Very high | 0.861 | 0.285 | 0.654 | 1.133 |
|  |  |  |  |  |
| Day of surgery: ref Tuesday |  |  |  |  |
| Fri | 0.863 | 0.385 | 0.619 | 1.204 |
| Mon | 0.754 | 0.023 | 0.591 | 0.962 |
| Sat | 1.600 | 0.410 | 0.523 | 4.897 |
| Sun | 0.541 | 0.226 | 0.200 | 1.463 |
| Thu | 1.182 | 0.217 | 0.906 | 1.540 |
| Wed | 1.092 | 0.463 | 0.863 | 1.381 |
|  |  |  |  |  |
| Provider region: East of England |  |  |  |  |
| East Midlands | 0.542 | 0.531 | 0.080 | 3.676 |
| London | 2.582 | 0.072 | 0.920 | 7.245 |
| North East | 1.968 | 0.339 | 0.492 | 7.878 |
| North West | 7.814 | <0.001 | 2.735 | 22.319 |
| South East | 1.109 | 0.832 | 0.424 | 2.905 |
| South West | 0.655 | 0.399 | 0.245 | 1.752 |
| Wales | 39.377 | <0.001 | 5.045 | 307.319 |
| West Midlands | 1.709 | 0.439 | 0.440 | 6.641 |
| Yorkshire and The Humber | 2.272 | 0.260 | 0.545 | 9.478 |
|  |  |  |  |  |
| Rurality: ref rural |  |  |  |  |
| urban | 1.037 | 0.425 | 0.948 | 1.134 |

Table S8b Prolonged LOS, imputed datasets, mixed effects logistic regression, Odds Ratio reported, N= 51,254

|  | OR | P value | 95 CI | |
| --- | --- | --- | --- | --- |
| straight_distance | 1.001 | 0.048 | 1.000 | 1.003 |
| centered_age | 1.018 | <0.001 | 1.014 | 1.022 |
| centered_age2 | 1.000 | <0.001 | 1.000 | 1.000 |
| gender |  |  |  |  |
| Male sex | 1.159 | 0.002 | 1.053 | 1.275 |
|  |  |  |  |  |
| Ref: Abdominal-Lower GI, Complex /complex major |  |  |  |  |
| Abdominal-H,Complex/complex maj | 0.398 | <0.001 | 0.283 | 0.559 |
| Abdominal-H,major/not known | 0.284 | 0.116 | 0.059 | 1.368 |
| Abdominal-Lower GI,major/not known | 0.574 | 0.060 | 0.322 | 1.024 |
| Abdominal-Upper GI,Complex/complex maj | 0.185 | <0.001 | 0.132 | 0.260 |
| Abdominal-Upper GI,major/not known | 0.078 | 0.005 | 0.013 | 0.470 |
| Abdominal-Other,Complex/complex maj | 0.721 | 0.092 | 0.493 | 1.055 |
| Abdominal-Other,major/not known | 0.132 | 0.003 | 0.035 | 0.496 |
| Burns and Plastics,Complex/complex maj | 7.694 | <0.001 | 2.871 | 20.618 |
| Gynaecology,Complex/complex maj | 3.941 | <0.001 | 2.705 | 5.741 |
| Head and neck,Complex/complex maj | 0.427 | <0.001 | 0.265 | 0.687 |
| Head and neck,major/not known | 0.138 | 0.244 | 0.005 | 3.861 |
| Orthopaedics,Complex/complex maj | 1.538 | 0.015 | 1.088 | 2.175 |
| Spinal,Complex/complex maj | 1.989 | 0.055 | 0.986 | 4.011 |
| Spinal,major/not known | 3.195 | <0.001 | 2.153 | 4.740 |
| Thoracics,Complex/complex maj | 2.952 | <0.001 | 2.211 | 3.940 |
| Thoracics,major/not known | 2.333 | <0.001 | 1.827 | 2.979 |
| Urology,Complex/complex maj | 2.534 | <0.001 | 1.990 | 3.226 |
| Urology,major/not known | 1.081 | 0.613 | 0.801 | 1.458 |
| Vascular,Complex/complex maj | 0.943 | 0.710 | 0.691 | 1.286 |
| Not recorded,major/not known | 3.250 | 0.074 | 0.893 | 11.832 |
|  |  |  |  |  |
| Cancer diagnosis or in remission for <5 years : ref |  |  |  |  |
| Yes | 1.004 | 0.936 | 0.906 | 1.114 |
|  |  |  |  |  |
| Enhanced recovery: ref Yes |  |  |  |  |
| No | 1.069 | 0.361 | 0.926 | 1.235 |
|  |  |  |  |  |
| Preop-assessment: ref Yes |  |  |  |  |
| No | 1.902 | <0.001 | 1.448 | 2.497 |
|  |  |  |  |  |
| Sodium: ref Normal |  |  |  |  |
| Abnormal | 1.346 | 0.003 | 1.110 | 1.633 |
|  |  |  |  |  |
| Creatinine: ref Normal |  |  |  |  |
| Abnormal | 1.066 | 0.131 | 0.981 | 1.159 |
|  |  |  |  |  |
| Albumin: ref Normal |  |  |  |  |
| Abnormal | 1.283 | <0.001 | 1.140 | 1.444 |
|  |  |  |  |  |
| White cell count:ref Normal |  |  |  |  |
| Abnormal | 1.046 | 0.516 | 0.912 | 1.200 |
|  |  |  |  |  |
| Haemoglobin: ref Normal |  |  |  |  |
| Abnormal | 1.207 | <0.001 | 1.099 | 1.325 |
|  |  |  |  |  |
| Pulse rate: ref Normal |  |  |  |  |
| Abnormal | 0.963 | 0.525 | 0.859 | 1.081 |
|  |  |  |  |  |
| Cerebrovascular Disease: No |  |  |  |  |
| Yes | 1.002 | 0.985 | 0.848 | 1.184 |
|  |  |  |  |  |
| Dementia: No |  |  |  |  |
| Yes | 1.374 | 0.179 | 0.865 | 2.183 |
|  |  |  |  |  |
| Diabetes: ref No diabetes |  |  |  |  |
| T1 & HbA1C<=8.5 | 1.200 | 0.570 | 0.639 | 2.255 |
| T1 & HbA1C>8.5 | 1.670 | 0.155 | 0.824 | 3.385 |
| T2 & HbA1C<=8.5 | 1.024 | 0.709 | 0.903 | 1.162 |
| T2 & HbA1C>8.5 | 1.222 | 0.042 | 1.008 | 1.481 |
|  |  |  |  |  |
| ASA grade: ref 1&2 |  |  |  |  |
| 3-5 | 1.398 | <0.001 | 1.269 | 1.539 |
|  |  |  |  |  |
| NYHA: ref class I |  |  |  |  |
| II | 1.059 | 0.290 | 0.952 | 1.178 |
| III or IV | 1.119 | 0.312 | 0.900 | 1.391 |
|  |  |  |  |  |
| Bowel prep: ref No |  |  |  |  |
| Yes | 1.305 | <0.001 | 1.168 | 1.457 |
|  |  |  |  |  |
| Frailty: ref Not frail 1-4 |  |  |  |  |
| Mild 5 | 1.285 | 0.034 | 1.019 | 1.621 |
| Moderate 6-9 | 1.668 | <0.001 | 1.276 | 2.181 |
|  |  |  |  |  |
| BMI: Normal/high |  |  |  |  |
| Low | 1.231 | 0.196 | 0.898 | 1.687 |
| Very high | 0.963 | 0.701 | 0.795 | 1.167 |
|  |  |  |  |  |
| Smoking status: never smoked as ref |  |  |  |  |
| Current smoker | 1.145 | 0.035 | 1.010 | 1.299 |
| Ex-smoker | 1.051 | 0.229 | 0.969 | 1.140 |
|  |  |  |  |  |
| urgency_of_surgery |  |  |  |  |
| Expedited | 1.025 | 0.719 | 0.895 | 1.175 |
|  |  |  |  |  |
| Duration of surgery: ref <2 hours |  |  |  |  |
| 2-3 hours | 1.561 | <0.001 | 1.335 | 1.827 |
| >3 hours | 2.800 | <0.001 | 2.318 | 3.383 |
|  |  |  |  |  |
| DrEaMing: ref No |  |  |  |  |
| Yes | 0.415 | <0.001 | 0.361 | 0.478 |
|  |  |  |  |  |
| Post-op complications: ref Grade I |  |  |  |  |
| Grade II | 6.711 | <0.001 | 5.896 | 7.639 |
| Grade III and above | 35.534 | <0.001 | 29.686 | 42.534 |
|  |  |  |  |  |
| Pain recovery: ref None |  |  |  |  |
| Mild | 0.977 | 0.548 | 0.907 | 1.053 |
| Moderate | 1.054 | 0.372 | 0.939 | 1.182 |
| Severe | 0.974 | 0.701 | 0.850 | 1.116 |
| Unable to ascertain | 1.302 | 0.005 | 1.083 | 1.567 |
|  |  |  |  |  |
| Nasogastric tube postop: ref No |  |  |  |  |
| Yes | 1.722 | <0.001 | 1.496 | 1.983 |
|  |  |  |  |  |
| Day of surgery: ref Tuesday |  |  |  |  |
| Fri | 1.018 | 0.809 | 0.878 | 1.181 |
| Mon | 1.083 | 0.181 | 0.964 | 1.217 |
| Sat | 0.956 | 0.889 | 0.511 | 1.792 |
| Sun | 2.255 | 0.003 | 1.320 | 3.854 |
| Thu | 0.967 | 0.568 | 0.862 | 1.085 |
| Wed | 1.107 | 0.203 | 0.947 | 1.294 |
|  |  |  |  |  |
| Provider region: East of England |  |  |  |  |
| East Midlands | 1.613 | 0.018 | 1.084 | 2.398 |
| London | 1.362 | 0.086 | 0.958 | 1.938 |
| North East | 0.931 | 0.807 | 0.522 | 1.658 |
| North West | 1.645 | 0.004 | 1.176 | 2.300 |
| South East | 1.048 | 0.820 | 0.702 | 1.564 |
| South West | 1.262 | 0.218 | 0.871 | 1.828 |
| Wales | 1.211 | 0.298 | 0.844 | 1.738 |
| West Midlands | 1.215 | 0.373 | 0.792 | 1.862 |
| Yorkshire and The Humber | 1.191 | 0.451 | 0.756 | 1.876 |
|  |  |  |  |  |
| Rurality: ref rural |  |  |  |  |
| urban | 1.130 | 0.014 | 1.025 | 1.245 |

**S9 List of PQIP project team and collaborators**

#### List of PQIP project team:

James Durrand

Duncan Wagstaff

Aiman Al-Eryani

Dominic Olive

Adam Hunt

Eleanor Warwick

Jo Simpson

Jose Lourtie

Christine Taylor

S Ramani Moonesinghe

#### PQIP collaborators list

Amarjeet D Patil Emma Jackson Reshma Shanmugam

Jessica Southwell Sumayer Sanghera Anjum Ahmed-Nusrath

Garima Daga Joanne Hopkins Maria-Olghita Tritean

Anjum Ahmed-Nusrath Jane Griffiths Jisha Josph

Victoria Cunliffe Emma Ward Julie Logan

Cathy White Leah Reid Mark pulletz

Duncan Chambler Alice Roberts Katie Edwards

Jen Entwisle Vicki Waugh Anirudda Pai

Faye Benson Kelly Musson Emily Redman

Michelle Eve David Griffiths Erin Lester

Rachel Lorusso Peter Bye Salman Ahmad

Asad Zafar Khan Muhammad Kamal Asad Masood

Subhro Mitra Tracy Edmunds Angeline Mbuyisa

Caroline Reavley Deirdre Fottrell-Gould Jane Platt

Melissa Ryan Ayman Fahim Shivacharan Rudrappa

Unnathi Manampadi Roshini Wijenayake Ewa Prusak

Sejal Modasia Emma Jackson Ourania Stefa

Clare Byrne Erin Lester David Griffiths

Reshma Shanmugam Jessica Southwell Rob Wiltshire

Karen O’Toole Charlotte Humphrey Sudha Garg

Darylile Guledew Lee Yee Tee Andy Yuet Meng Ng

Zoe Ridgway Helen Langton Suprita Dewan

Charlotte Hunt Nikola Robinson Emma Marshall

Louisa Davies Nicholas Courtenay-Evans Francesca Wing

Isaac Brookman Catherine Kloppenborg Cherisse Ogbinar

Henry Lewith Chris Watson Anjana Mistry

Helen Morgan Tim Hughes Emma Clarey

Kevin O'Reilly Arav Gupta Chloe Bussell

Matthew Evans Chris Jones Natalia Michalak-Glinska

Hafsana Ali Ben Morrison Elouise Helme

Leigh Kelliher Paula Carvelli Michael Nesbitt

David Evans Elena Williams Samantha Ley

Jennifer Morrison Mohan Ranganathan Angela Day

Judy Shirley Alistair Wilson Bridget Campbell

Richard Ramsaran Vicki Waugh Jen Entwistle

Katie Edwards Alice Roberts Sam Pickard

Michael Ho Marta Blanco Cabana Aminah Ahmad

Joesph Hogan Andrews Cowan-Williams Sylvia Martin

Ayat Mohamed Sonia Mason Afnan Mahmoud

Ayesha Banu Nosaiba Ezzelarab Hassan Abdelhalim

Sheikh Surumi Fraser Kenny Amanda Jones

Annette Bolger Angela Pye Bethan Roberts

Caroline Lees Esther Garrod Joanne Lewis

Jennifer Davies Karen Williams Lois Bexon

Oluwatoyin Idowu Pauline Burford Rachel Manley

Sharon Harland Tracy Rich Victoria Garvey

Zuzana Probier VeGeetanjali Verma Diane Armstrong

Katherine Davidsonnkat Sundaram Rachel Mutch Lynn Sutcliffeeshnie Govender

Hinna Zahur Abegail Alvaran Marta Correia

Vanessa Machado Bridget Oduro Muhammad Farhan Rasheed

Adam Eddie Laura Cheverst Felicity Pilkington

Alex Gerard Rhiannon Eastwood Joe Sullivan

Kieran Slade Marten Lau Tilly Boa

Will Southall Keri Joslyn Oliver George

Lewis Moore Namitha Jayaprabhu Lily Rietbergen

Oscar Atkinson Lea Habib Alexander Palmer

Sofia Farina Elodie Oweis Kavya Pillai

Cyrille Cabaret Tilly Gibson-Watt Alexandra Matson

Priyakam Chowdhury Jamie Gonzales Amrinder Sayan

Monica Popescu Amirdhabashini Gnanasoruban Jikson Kainadathuparambil Johnson
